# Mode of transport, genetic susceptibility, and incidence of coronary heart disease

**DOI:** 10.1186/s12966-023-01484-4

**Published:** 2023-07-04

**Authors:** Zhu Liduzi Jiesisibieke, Jenna Panter, Mengyao Wang, Shiu Lun Au Yeung, Shan Luo, Haeyoon Jang, Eric Yuk Fai Wan, Soren Brage, Youngwon Kim

**Affiliations:** 1 School of Public Health, The University of Hong Kong Li Ka Shing Faculty of Medicine, Room 301D 3/F, Jockey Club Building for Interdisciplinary Research, 5 Sassoon Road, Pokfulam, Hong Kong SAR, China; 2 MRC Epidemiology Unit, University of Cambridge School of Clinical Medicine, Institute of Metabolic Science, Cambridge Biomedical Campus, Box 285, Cambridge, CB2 0QQ Cambridgeshire UK; 3 UKCRC Centre for Diet and Activity Research (CEDAR), University of Cambridge, Cambridge, CB2 0QQ UK; 4 School of Public Health, Li Ka Shing Faculty of Medicine, The University of Hong Kong, 1/F, Patrick Manson Building, 7 Sassoon Road, Pokfulam, Hong Kong SAR, China; 5 Department of Family Medicine and Primary Care, The University of Hong Kong Li Ka Shing Faculty of Medicine, 3/F, Ap Lei Chau Clinic, 161 Main Street, Ap Lei Chau, Hong Kong SAR, China; 6 Department of Pharmacology and Pharmacy, The University of Hong Kong Li Ka Shing Faculty of Medicine, Laboratory Block LKS Faculty of Medicine, General Office, L02-56 2/F, , 21 Sassoon Road, Pokfulam, Hong Kong SAR, China

**Keywords:** Active transport, Genetic susceptibility, Coronary heart disease, UK Biobank

## Abstract

**Background:**

Car use has been associated with higher risk of coronary heart disease (CHD). However, whether the associations of transport modes with CHD vary by genetic susceptibility to CHD are unknown. This study aims to investigate the associations of genetic susceptibility and modes of transport with incidence of CHD.

**Methods:**

We included 339,588 white British participants from UK Biobank with no history of CHD or stroke at baseline or within two years of follow-up (52.3% in work). Genetic susceptibility to CHD was quantified through weighted polygenic risk scores derived from 300 single-nucleotide polymorphisms related to CHD risk. Categories of transport mode included exclusive car use and alternatives to the car (e.g., walking, cycling and public transport), separately for non-commuting (e.g., getting about [*n*=339,588] excluding commuting for work), commuting (in the sub-set in work [*n*=177,370] who responded to the commuting question), and overall transport (transport mode for both commuting and non-commuting [*n*=177,370]). We used Cox regression with age as the underlying timescale to estimate hazard ratios (HR) of CHD (*n*=13,730; median 13.8-year follow-up) and tested the interaction between genetic susceptibility and travel modes with adjustment for confounders.

**Results:**

Compared to those using alternatives to the car, hazards of CHD were higher for exclusive use of cars for overall transport (HR: 1.16, 95% confidence interval (CI): 1.08-1.25), non-commuting (HR: 1.08, 95% CI: 1.04-1.12) and commuting (HR: 1.16, 95% CI: 1.09-1.23), after adjusting for confounders plus genetic susceptibility. HRs of CHD were 1.45 (95% CI: 1.38-1.52) and 2.04 (95% CI: 1.95-2.12) for the second and third tertile of genetic susceptibility to CHD, respectively, compared to the first. There was, in general, no strong evidence of interactions between genetic susceptibility and categories of overall, non-commuting and commuting transport. Estimated 10-year absolute risk of CHD was lower for the alternatives to the car across strata of genetic susceptibility, compared with exclusive use of cars for overall, non-commuting and commuting transport.

**Conclusion:**

Exclusive use of cars was associated with a relatively higher risk of CHD across all strata of genetic susceptibility. Using alternatives to the car should be encouraged for prevention of CHD for the general population including individuals at high genetic risk.

**Supplementary Information:**

The online version contains supplementary material available at 10.1186/s12966-023-01484-4.

## Introduction

Coronary heart disease (CHD) is a major public health burden, causing 9.14 million deaths and 182 million disability-adjusted life years globally [1]. Development of CHD is attributable to both lifestyle and genetic traits [2, 3]. Physical activity is a key lifestyle behavioural predictor of CHD [4]. However, levels of physical activity have declined substantially over the past few decades, primarily driven by changes in technology and transport [5]. As such, active modes of commuting and non-commuting transport can play an integral role in the accumulation of daily activity [6]. For example, transport-related physical activity can account for over 11% of overall daily physical activity or daily physical activity energy expenditure, and passive transport for 6% of daily sedentary time among adults [7]. According to a survey conducted by Trades Union Congress, the average commuting time in the UK was 59 minutes a day in 2018 [8]. Previous research has found that car use, as opposed to alternatives to the car (walking, cycling and public transport), was associated with higher risks of cardiovascular disease mortality [9] and CHD [10]. However, the World Health Organization guidelines on physical activity and sedentary behaviour specifically indicate the need for further research on the health impacts of different types of transportation, which is a major target domain for promoting physical activity [6]. Of public health strategies that promote physical activity, interventions employing a multi-level and multi-component approach with support from public health and transport policies have the potential for promoting more active modes of travel, and thereby, generating small but sustainable population-level changes in physical activity [11–13]. As such, promoting active transport as a means of increasing physical activity as well as preventing cardiovascular disease is currently of public health relevance.

Evidence suggests that up to 70% of the heritability of CHD can be accounted for by known single nucleotide polymorphisms (SNP) [14]. Polygenic risk scores (PRS) derived from a multitude of genetic variants for CHD have the potential for stratifying individuals by genetic risk, thereby making it possible to identify a subset of individuals at high genetic risk of CHD [15]. While previous research has investigated the interplay of lifestyle, behaviour-related traits and genetic risk of CHD [16–20], there is currently limited understanding of the role that active transport could play in CHD prevention when genetic risk of CHD is taken into consideration. There can also be different implications of using an inactive mode of transport (e.g., cars) for the risk of CHD across the spectrum of genetic risk of CHD. The purpose of this study was, therefore, to examine whether associations of modes of transport with incident CHD are independent of and vary depending on genetic susceptibility to CHD using a large prospective cohort study.

## Methods

### Study design and participants

**Fig. 1:**
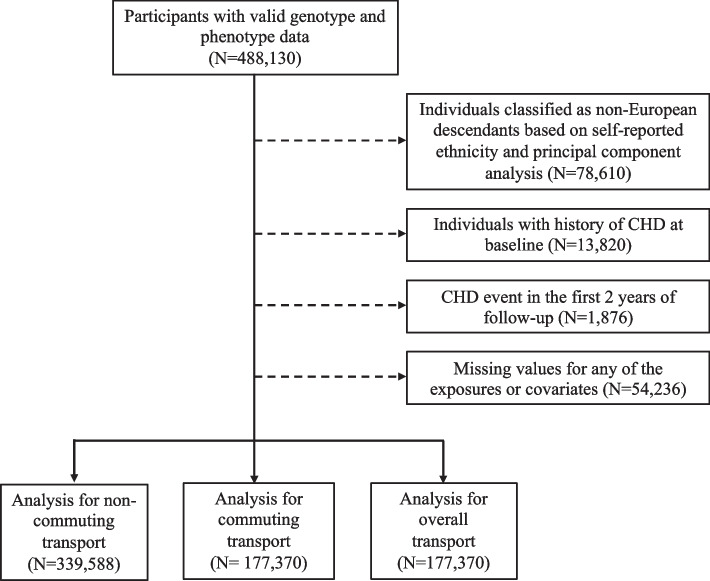
Participants’ flow chart

The UK Biobank is a large-scale prospective community-based cohort study including more than half a million UK adults aged 40-69 years upon recruitment who lived within 25 miles of 1 of 22 assessment centres across England, Scotland and Wales. At baseline (from 2006 to 2010), a comprehensive series of variables were collected including assessments of lifestyle behavioural variables (including transport mode used, smoking, alcohol, physical activity and diet) and socio-demographic indicators at baseline, through self-administered touch-screen questionnaires, measurements of physical characteristics (height, weight, grip strength, etc.) and collection of biological samples (blood, urine and saliva) [21]. The protocol of UK Biobank is described in more detail elsewhere [22, 23]. UK Biobank was approved by the Northwest Multicentre Research Ethics Committee (reference no. 11/ NW/0382) and all participants provided written informed consent prior to participation. This study included 333,426 white British individuals (based on self-reported ethnicity combined with principal component analysis of genotype data), after excluding prevalent cases of CHD and stroke and those experiencing an event in the first two years of follow-up, as well as participants with any missing data for any of the covariates (See Fig. 1).

### Exposures

#### Polygenic risk scores for CHD

In the UK Biobank project, genotyping was conducted on all participants with two types of genotyping arrays, UK BiLEVE and UK Biobank Axiom [24]. We included 300 genome-wide significant and uncorrelated SNPs (at a false discovery rate of 5%) [25, 26] known to be associated with risk of CHD (Supplemental Table 1 and Supplemental Figure 1). Weighted PRS for CHD were calculated that represent each individual’s genetic susceptibility to CHD. Specifically, the calculation of each individual’s weighted PRS was based on the sum of the products of risk-increasing alleles for each of 300 SNPs and its respective known effect estimates [25]. Three categories of genetic risk of CHD were generated according to the tertiles of PRS: low, medium, and high genetic risk.

#### Mode of transport

In UK Biobank, the information of participants’ mode of transport was collected at baseline through questionnaires. Participants were asked to response to two transport-related questions: “What types of transport do you use to get to and from work?” (indicative of transport for commuting), and "In the last 4 weeks, which forms of transport have you used most often to get about? (Not including any journeys to and from work)” (indicative of transport for non-commuting). A total of 6 response options were provided for each of these two questions: car/motor vehicle, walk, public transport, cycle, “none of the above” and “prefer not to answer” (See Supplemental Figure 2). For each question, participants were allowed to choose more than one option, which precluded the possibility of generating multiple distinctive categories of mode of transport in the same analysis. We, therefore, created two types of travel mode for commuting and non-commuting transport separately: exclusive use of cars and alternatives to the car (derived based on exclusive use of either walking, cycling or public transport alone, or in combination with use of cars). Similarly, we generated three types of travel mode for overall transport: exclusive use of cars, mixed transport mode (i.e., active transport for commuting and exclusive use of cars for non-commuting; or active transport for non-commuting and exclusive use of cars for commuting) and alternatives to the car. Participants who only reported “none of the above” or “prefer not to answer” were excluded in the present analysis. No information on trip frequency and distance was reported for each mode of transport.

### Outcome

We used Codes of International Classification of Diseases (ICD) (ICD-9: 410-412, ICD-10: I21-I24, I25.2) and Office of Population Censuses and Surveys Classification of Interventions and Procedures Version 4 (OPCS-4) classifications [OPCS-4: K40-K46, K49, K50.1, K50.2, K50.4, K75] to identify CHD cases based on hospital admission records, operation procedures and death records. Incident CHD was defined as the first occurrence of CHD events accrued over a 13.8-year median follow-up (interquartile range: 13.1-14.5 years); last censored on December 9, 2022, for participants in England and Wales, and December 19, 2022, for participants in Scotland. A total of 13,730 incident CHD cases were included in the present analysis.

### Confounders

Models were adjusted for the following variables (chosen based on established knowledge according to the established practice [27]) that may serve as confounders (not acting as mediators) [28] in the associations of mode of transport with CHD incidence: age (underlying timescale), sex, body mass index (kg/m^2^), smoking status (never, previous, current), Townsend Deprivation Index (a composite score of employment, car ownership, home ownership and household overcrowding indicating area-specific deprivation), alcohol intake (never, previous, <3 times er week, current [more than 3 times per week]), salt intake (never/rarely, sometimes, usually, always), oily fish consumption (never, less than once per week, once per week, more than once per week), coffee intake (cups per day), fruit and vegetable intake (score ranging from 0-4 based on fresh/dried fruit intake and raw/cooked vegetable intake), processed/red meat intake (days per week), blood-pressure-lowering medication use, cholesterol-lowering medication use, TV viewing (hours/day in 1-hour increment), computer use (hours/day in 1-hour increment), sleep (hours per day in 1-hour increment), walking for pleasure (minutes per day), light do-it-yourself activities (minutes per day), heavy do-it-yourself activities (minutes per day), strenuous sports (minutes per day), other exercises (such as swimming, cycling, keeping fit; minutes per day), binary genotyping array type and the first ten principal components of genetic ancestry (to adjust for population stratification) [29].

### Statistical analyses

Cox regression models using age as the underlying timescale were used to estimate the associations between modes of transport and incident CHD with adjustment for all confounders; all models were fit after excluding incident CHD cases accrued over the first two years of follow-up and using cluster-robust standard errors [30] to adjust for the 2^nd^-degree genetic relatedness (defined as kinship coefficients between 0.0442 and 0.0884) [31]. Models using PRS as exposure were adjusted for sex, genotyping array type and the first ten principal components of genetic ancestry. For joint association analyses, we generated 6 joint groups based on the combination of tertiles of PRS and two types of transport (3 genetic risk categories × 2 types of transport) for non-commuting and commuting transport separately; and 9 joint groups (3 genetic risk categories × 3 types of transport) for overall transport. We tested both multiplicative and additive interactions between transport modes and PRS for incident CHD in models adjusted for all confounders. There were no covariates with high multicollinearity (Supplemental Table 2). Cumulative hazards of CHD across all ages were plotted for categories of mode of transport and PRS. We estimated 10-year absolute risk of CHD for each category of PRS and mode of transport. An interaction directed acyclic graph is provided in Supplemental Figure 3. We performed five sensitivity analyses: (1) excluding incident CHD events accrued over the first four years of follow-up to further address potential reverse causation, (2) retaining 1 participant randomly selected from each set of genetically related individuals (at 2^nd^-degree) to address any potential bias arising from the misclassification of genetically defined family membership, (3) using a weighted polygenic risk score calculated using 46 lead SNPs (from 46 loci) genome-wide significant at a *p*-value of 5×10^-8^ and in low linkage disequilibrium according to an *r*^2^ value of <0.001 (Supplemental Figure 4), (4) using a multiple imputation method to deal with missing covariates (assuming data missing at random; Supplemental Table 3), and (5) using CHD follow-up data censored on March 1st, 2020 to remove the potential possibility that some CHD cases were not adjudicated due to participants’ fear for visiting hospitals during the Coronavirus disease 2019 (COVID-19) pandemic. Analyses were performed using Stata/MP Version 17.0 (StataCorp LP, College Station, TX).

## Results

Table 1 presents the characteristics of individuals for overall transport, non-commuting transport, and commuting transport. Approximately 37% of individuals (*N*=66,072) self-reported exclusive use of cars for overall transport; about 39% of individuals (*N*=132,211) and 65% of individuals (*N*=115,915) used cars exclusively for non-commuting and commuting, respectively. Mean age and proportions of medication use were higher in participants included in the analysis for non-commuting transport than in those in the analyses for overall and commuting transport.

**Table 1:** Characteristics of individuals overall and within three categories of commuting and non-commuting

Variables	All (*N*=339,588)	Overall transport (*N*=177,370)			Non-commuting transport (*N*=339,588)		Commuting transport (*N*=177,370)	
		Exclusive use of cars (*N*=66,072)	Mixed transport (*N*=62,917)	Alternatives to the car (*N*= 48,381)	Exclusive use of cars (*N*= 132,211)	Alternatives to the car (*N*= 207,377)	Exclusive use of cars (*N*= 115,915)	Alternatives to the car (*N*= 61,455)
Age, years	56.7 (8.0)	52.5 (6.8)	52.2 (6.8)	52.1 (6.9)	56.0 (7.9)	57.2 (8.1)	52.4 (6.8)	52.1 (6.8)
Sex, n (%)								
Men	154,007 (45.3)	33,339 (50.6)	30,788 (48.9)	21,817 (45.1)	62,235 (47.1)	91,772 (44.1)	58,072 (50.1)	27,872 (45.4)
Women	185,581 (54.7)	32,733 (49.5)	32,129 (51.1)	26,564 (54.9)	69,976 (52.9)	115,605 (55.9)	57,843 (49.9)	33,583 (54.6)
Body mass index, kg/m^2^	27.2 (4.6)	27.6 (4.7)	27.1 (4.4)	26.5 (4.5)	27.6 (4.7)	26.9 (4.5)	27.4 (4.6)	26.6 (4.5)
Smoking status, %								
Never	190,497 (56.1)	37,780 (57.2)	37,609 (59.8)	28,701 (59.3)	73,025 (55.2)	117,472 (56.6)	67,313 (58.1)	36,744 (59.8)
Previous	117,680 (34.7)	21,122 (32.0)	19,636 (31.2)	14,791 (30.6)	46,401 (35.1)	71,279 (34.4)	36,849 (31.7)	18,700 (30.4)
Current	31,411 (9.2)	7,170 (10.8)	5,672 (9.0)	4,889 (10.1)	12,785 (9.7)	18,626 (9.0)	11,753 (10.1)	5,978 (9.7)
Townsend Deprivation Index	-1.7 (2.8)	-2.1 (2.6)	-1.9 (2.6)	-0.7 (3.1)	-2.1 (2.6)	-1.4 (3.0)	-2.0 (2.6)	-0.9 (3.1)
Alcohol Consumption Status								
Never	9,804 (2.9)	1,291 (1.9)	1,225 (2.0)	1,101 (2.3)	3,538 (2.7)	6,266 (3.0)	2,242 (1.9)	1,351 (2.2)
Previous	10,235 (3.0)	1,513 (2.3)	1,275 (2.0)	1,377 (2.8)	3,691 (2.8)	6,544 (3.2)	2,506 (2.2)	1,659 (2.7)
(<3times per week)	162,083 (47.7)	33,170 (50.2)	31,464 (50.0)	23,573 (48.7)	63,348 (47.9)	98,735 (47.6)	58,151 (50.2)	30,056 (48.9)
Current (more than 3times per week)	157,466 (46.4)	30,098 (45.6)	28,953 (46.0)	22,330 (46.2)	61,634 (46.6)	95,832 (46.2)	53,016 (45.7)	28,365 (46.2)
Salt-adding behaviour								
Never/rarely	194,530 (57.3)	36,367 (55.1)	36,714 (58.4)	29,075 (60.1)	72,711 (55.0)	121,819 (58.7)	65,414 (56.4)	36,742 (59.8)
Sometimes	93,933 (27.7)	18,916 (28.6)	17,573 (28.0)	13,242 (27.4)	37,316 (28.2)	56,617 (27.3)	32,874 (28.4)	16,857 (27.4)
Usually	37,591 (11.0)	7,660 (11.6)	6,519 (10.3)	4,546 (9.4)	16,005 (12.1)	21,586 (10.4)	12,817 (11.1)	5,908 (9.6)
Always	13,534 (4.0)	3,129 (4.7)	2,111 (3.3)	1,518 (3.1)	6,179 (4.7)	7,355 (3.6)	4,810 (4.1)	1,948 (3.2)
Oily fish consumption								
Never	34,684 (10.2)	8,425 (12.7)	7,192 (11.4)	5,755 (11.9)	14,217 (10.8)	20,467 (9.9)	14,135 (12.2)	7,237 (11.8)
< Once per week	113,837 (33.5)	25,580 (38.7)	23,424 (37.3)	16,915 (35.0)	47,911 (36.1)	66,144 (31.9)	43,736 (37.7)	22,180 (36.1)
Once per week	131,321 (38.7)	23,105 (35.0)	22,185 (36.8)	17,840 (36.8)	49,719 (37.6)	81,602 (39.3)	41,661 (36.0)	22,469 (36.5)
> Once per week	59,746 (17.6)	8,962 (13.6)	9,116 (14.5)	7,871 (16.3)	20,582 (15.6)	39,164 (18.9)	16,383 (14.1)	9,566 (15.6)
Coffee intake (cups per day)	2.1 (2.0)	2.2 (2.3)	2.1 (2.1)	2.0 (2.0)	2.1 (2.1)	2.0 (2.0)	2.2 (2.2)	2.1 (2.0)
Fruit and vegetable intake (score ranging from 0-4 based on fresh/dried fruit intake and raw/cooked vegetable intake)	1.6 (1.1)	1.4 (1.1)	1.5 (1.1)	1.6 (1.2)	1.5 (1.1)	1.7 (1.2)	1.5 (1.1)	1.6 (1.1)
Red meat intake, days/week (average)	0.9 (0.5)	0.9 (0.5)	0.9 (0.5)	0.8 (0.5)	0.9 (0.5)	0.9 (0.5)	0.9 (0.5)	0.8 (0.5)
Hypertension medication use, %	62,882 (18.5)	8,764 (13.3)	7,456 (11.9)	5,350 (11.1)	24,073 (18.2)	38,809 (18.7)	14,835 (12.8)	6,735 (10.9)
Cholesterol-lowering medication use, %	49,159 (14.5)	6,224 (9.4)	5,127 (8.2)	3,697 (7.6)	18,908 (14.3)	30,251 (14.6)	10,369 (9.0)	4,679 (7.6)
TV-viewing, hours/day	2.7 (1.5)	2.5 (1.3)	2.4 (1.3)	2.2 (1.4)	2.8 (1.5)	2.7 (1.6)	2.5 (1.3)	2.2 (1.4)
Computer use, hours/day	1.0 (1.3)	1.1 (1.4)	1.0 (1.2)	1.0 (1.3)	1.1 (1.3)	1.0 (1.2)	1.0 (1.3)	1.0 (1.3)
Sleep, hours/day	7.2 (1.0)	7.0 (0.9)	7.1 (0.9)	7.0 (0.9)	7.2 (1.0)	7.2 (1.0)	7.0 (0.9)	7.0 (0.9)
total walk for pleasure (minutes per day)	15.5 (23.1)	9.3 (15.5)	14.0 (20.2)	13.0 (19.4)	11.2 (18.6)	18.2 (25.2)	11.9 (18.4)	12.2 (18.6)
total light DIY (minutes per day)	10.8 (25.3)	8.3 (21.2)	9.7 (22.5)	7.5 (17.3)	10.0 (24.7)	11.3 (25.6)	9.1 (22.3)	7.5 (17.4)
total heavy DIY (minutes per day)	6.9 (20.5)	6.8 (22.0)	6.8 (20.1)	4.7 (14.1)	7.2 (21.9)	6.8 (19.5)	7.0 (21.6)	4.7 (14.3)
total strenuous sports (minutes per day)	2.4 (10.0)	2.7 (9.7)	3.0 (10.3)	3.3 (11.5)	2.4 (9.6)	2.4 (10.2)	2.8 (10.0)	3.3 (11.2)
total other exercises (minutes per day)	9.6 (17.6)	8.3 (15.0)	9.0 (15.4)	10.7 (18.2)	9.1 (16.8)	10.0 (18.1)	8.7 (15.3)	10.3 (17.6)
Polygenic risk scores for CHD	17.3 (0.6)	17.3 (0.6)	17.3 (0.6)	17.3 (0.6)	17.3 (0.6)	17.3 (0.6)	17.3 (0.6)	17.3 (0.6)

*Note*: Values are means (standard deviations) or percentages, unless otherwise indicated

Table 2 summarises the results of the associations between transport modes and incident CHD. Compared with using alternatives to the car for transport, exclusive use of cars and mixed transport mode for overall transport were associated with 16% (hazard ratio (HR): 1.16, 95% confidence interval (CI): 1.08-1.25) and 11% higher hazards of CHD (HR: 1.11, 95% CI: 1.03-1.19), respectively, after adjusting for all confounders and PRS for CHD. Participants who used cars exclusively for either non-commuting (HR: 1.08, 95% CI: 1.04-1.12) or commuting (HR: 1.16, 95% CI: 1.09-1.23) had higher CHD hazards compared with those who used the alternatives after adjustment for PRS as well as confounders. These findings were, in general, similar in sensitivity analyses, as presented in Supplemental Tables 4, 5, 6, 7 and 8.

**Table 2:** Associations of mode of transport and genetic susceptibility with incident coronary heart disease (CHD)

**Comparison**	**Number of participants**	**Number of cases**	**Crude incident rate per 100,000-person years**	**Hazard ratio of CHD (95% confidence interval)**	
				**Model 1**	**Model 2**
**Overall transport (*N*=177,370)**					
Alternatives to the car (Reference)	48,381	1,218	183.5	1.00 (Reference)^a^	1.00 (Reference)
Mixed transport mode	62,917	1,915	221.4	1.19 (1.11, 1.28)	1.11 (1.03, 1.19)
Exclusive use of cars	66,072	2,322	255.9	1.35 (1.26, 1.45)	1.16 (1.08, 1.25)
**Non-commuting transport (*N*=339,588)**					
Alternatives to the car (Reference)	207,377	8,170	289.1	1.00 (Reference)^a^	1.00 (Reference)
Exclusive use of cars	132,211	5,560	307.9	1.15 (1.12, 1.19)	1.08 (1.04, 1.12)
**Commuting transport (*N*=177,370)**					
Alternatives to the car (Reference)	61,455	1,537	182.2	1.00 (Reference)^a^	1.00 (Reference)
Exclusive use of cars	115,915	3,918	246.1	1.31 (1.23, 1.39)	1.16 (1.09, 1.23)
**Tertiles of genetic risk (*N*=339,588)**					
Low (Reference)	113,105	3,118	201.0	1.00 (Reference)^b^	-
Medium	113,22	4,478	289.8	1.45 (1.38, 1.52)	-
High	113,261	6,134	399.5	2.04 (1.95, 2.12)	-

*Notes*:

Model 1^a^: adjusted for age (underlying timescale)

Model 1^b^: adjusted for age (underlying timescale), sex, genotyping array type and the first ten principal components of genetic ancestry

Model 2: adjusted for age (underlying timescale), sex, body mass index, smoking (never, previous, current), alcohol intake (never, previous, currently <3 times/week, currently ≥3 times/week), salt intake (never/rarely, sometimes, usually, always), oily fish intake (never, <once per week, once per week, >once per week), coffee intake (cups per day), fruit and vegetable intake (a composite score based on fresh/dried fruit intake and raw/cooked vegetable intake), processed/red meat intake (days per week), Townsend Deprivation Index (an indicator of area-based socioeconomic status), sleep (≤5, 6, 7, 8 and ≥9hours per day), total leisure-time physical activity (minutes per day; based on walking, non-walking moderate physical activity and non-walking vigorous physical activity), blood-pressure-lowering medication use, cholesterol-lowering medication use, polygenic risk scores, genotyping array type and the first ten principal components of genetic ancestry

**Fig. 2:**
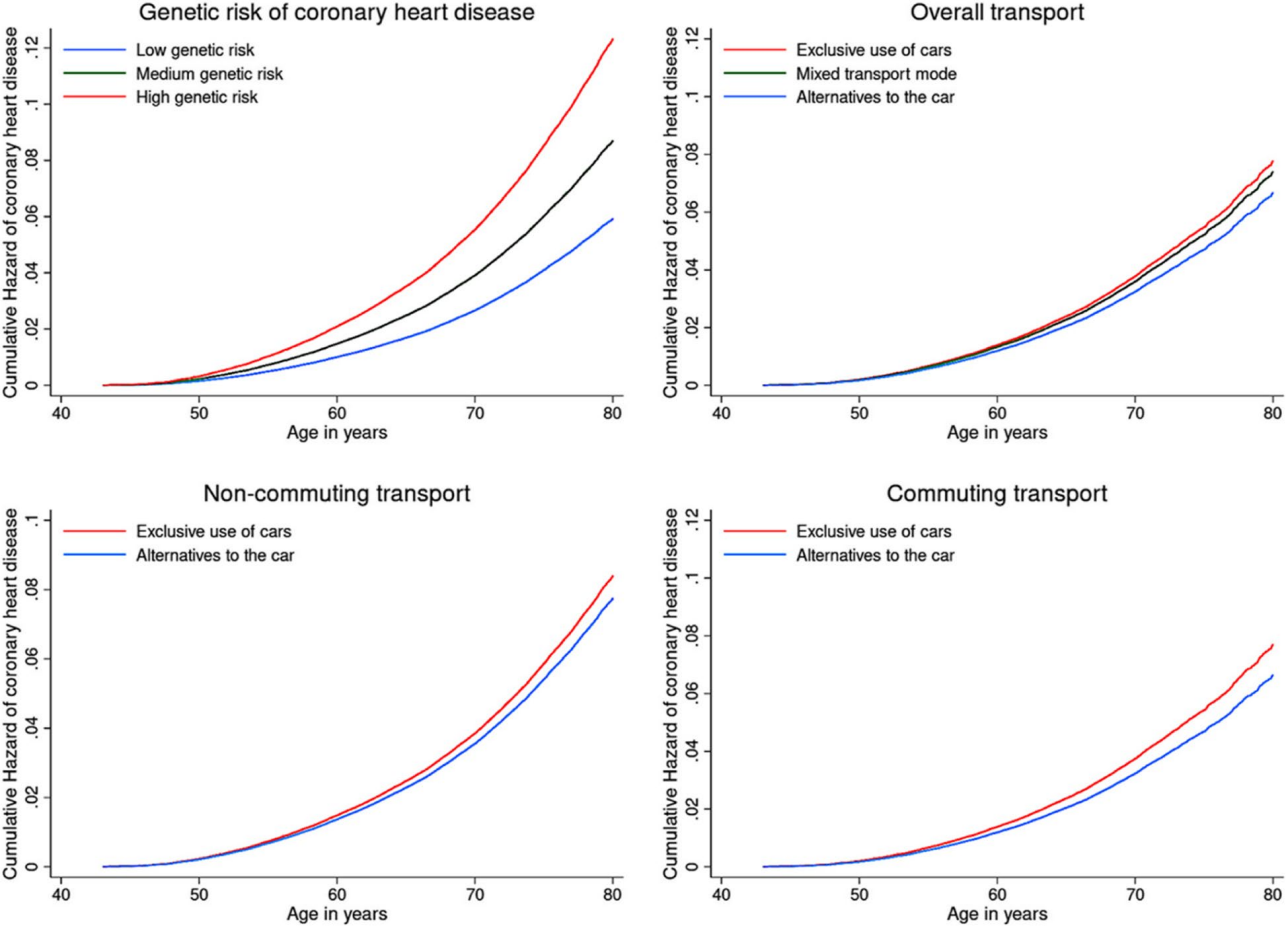
Cumulative hazard of coronary heart disease (CHD) for each category of transport mode and genetic risk across age ranges. Notes: Cumulative hazard of coronary heart disease for transport modes was adjusted for age (underlying timescale), sex, body mass index, smoking (never, previous, current), alcohol intake (never, previous, currently <3 times/week, currently ≥3 times/week), salt intake (never/rarely, sometimes, usually, always), oily fish intake (never, <once per week, once per week, >once per week), coffee intake (cups per day), fruit and vegetable intake (a composite score based on fresh/dried fruit intake and raw/cooked vegetable intake), processed/red meat intake (days per week), Townsend Deprivation Index (a measurement of area-based socioeconomic status), sleep (≤5, 6, 7, 8 and ≥9hours per day), total leisure-time physical activity (min per day; based on walking, moderate physical activity and vigorous physical activity), antihypertensive medication use, anticholesterolemic medication use, antidiabetic medication use, genotyping array type and the first ten principal components of genetic ancestry. Cumulative hazard of coronary heart disease for genetic risk was adjusted for age (underlying timescale), sex, genotyping array type and the first ten principal components

In Figure 2, we present the cumulative hazards of CHD for categories of transport modes and genetic risk across the age range. For overall transport, commuting and non-commuting transport, participants exclusively using cars had higher levels of cumulative CHD hazards at all ages, compared with using alternative modes of transport. Compared with individuals with low genetic susceptibility, those with medium and high genetic susceptibility had higher risk of CHD, after adjusting for age (underlying timescale), sex, genotyping array type and the first ten principal components of genetic ancestry.

**Fig. 3:**
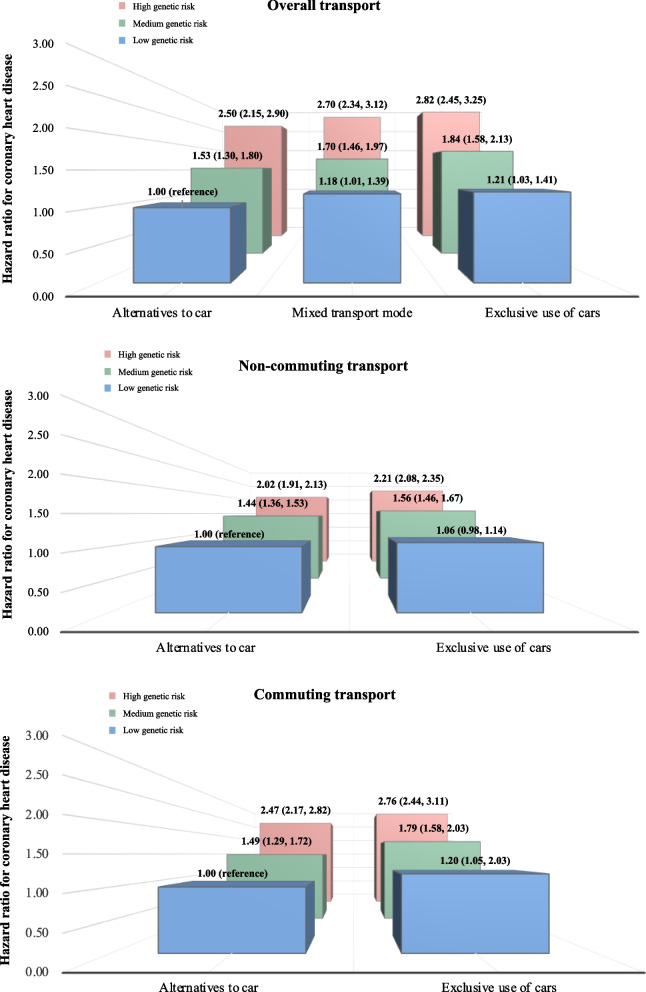
Joint associations of categories of transport modes and genetic susceptibility with incident coronary heart disease. Hazard ratios of coronary heart disease along with the corresponding 95% confidence intervals were presented. Model was adjusted for age (underlying timescale), sex, body mass index, smoking (never, previous, current), alcohol intake (never, previous, currently <3 times/week, currently ≥3 times/week), salt intake (never/rarely, sometimes, usually, always), oily fish intake (never, <once per week, once per week, >once per week), coffee intake (cups per day), fruit and vegetable intake (a composite score based on fresh/dried fruit intake and raw/cooked vegetable intake), processed/red meat intake (days per week), Townsend Deprivation Index (an indicator of area-based socioeconomic status), sleep (≤5, 6, 7, 8 and ≥9hours per day), total leisure-time physical activity (minutes per day; based on walking, non-walking moderate physical activity and non-walking vigorous physical activity), blood-pressure-lowering medication use, cholesterol-lowering medication use, glucose-lowering medication use, genotyping array type and the first ten principal components of genetic ancestry. For overall transport, *p*-value for multiplicative interaction=0.475; and *p*-value for additive interaction=0.045. For non-commuting, *p*-value for multiplicative interaction=0.427; and *p*-value for additive interaction=0.003. For commuting, *p*-value for multiplicative interaction=0.280; and *p*-value for additive interaction=0.067

Figure 3 summarises the joint associations of transport modes and categories of genetic susceptibility with incident CHD. For overall transport, exclusive use of cars in combination with low (HR:1.21, 95% CI: 1.03-1.41), medium (HR:1.84, 95% CI: 1.58-2.13) and high genetic risk (HR: 2.82, 95% CI: 2.45-3.25) of CHD was associated with relatively higher hazards of CHD, compared with using alternatives to the car (Supplemental Table 9); however, there was no multiplicative interaction between transport mode and genetic susceptibility (*p*-value=0.475) while there was evidence of additive interaction (*p*-value=0.045). Similar patterns of associations were observed for non-commuting and commuting, with generally higher hazards of CHD for exclusive use of cars combined with each level of genetic risk. While there was evidence of additive interaction (*p*-value = 0.003) for non-commuting, no evidence was observed for multiplicative interaction (*p*-value = 0.427) for non-commuting, and for both multiplicative (*p*-value = 0.280) and additive interactions (*p*-value = 0.067) for commuting.

**Fig. 4:**
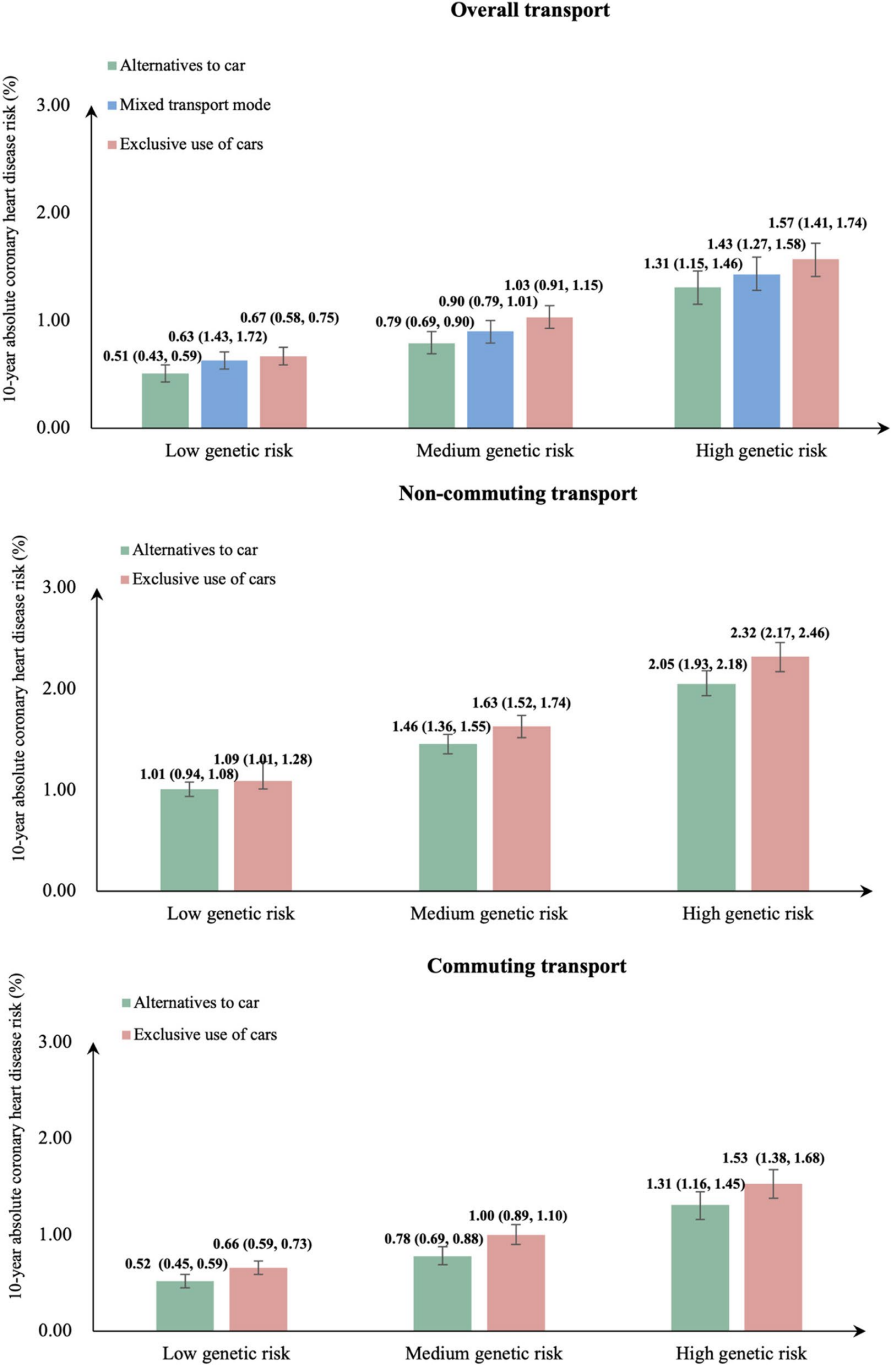
10-year absolute risk of coronary heart disease (CHD) for each category of transport mode across strata of genetic susceptibility to CHD. Note: Models were adjusted for age (underlying timescale), gender, genotyping array type and the first ten principal components of genetic ancestry. The 95% confidence intervals were shown in the form of error bars

Figure 4 shows estimates of 10-year absolute risk of CHD adjusted for age, sex, and genotyping array type and the first ten principal components of genetic ancestry. Participants who used alternatives to the car had lower 10-year absolute CHD risk compared with those who used cars exclusively within each level of genetic susceptibility.

## Discussion

This study is the first to investigate the interplay of different modes of transport and genetic susceptibility to CHD for incident CHD. Our results found that, irrespective of genetic susceptibility to CHD, exclusively using cars for any travel purpose was associated with a higher risk of CHD compared with using alternatives to the car. Notably, risk of CHD was, in general, lower (albeit wide and overlapping 95%CIs in some comparisons) for use of alternatives to the car than for exclusive use of cars in the full-sample analysis adjusted for genetic risk of CHD, and in analyses stratified by genetic risk. These findings shed new light on the potential role of active transport in prevention of cardiovascular events, and the importance of promoting more active transport for all individuals, including those whose genetic risk of CHD is high. As such, there will be a substantial public health benefit of shifting to more active modes of transport, particularly in individuals who have higher genetic risk of CHD.

Previous research has reported on the interplay of healthy lifestyle behaviours including high physical activity and fitness, and less screen-based sedentary time [2, 16, 17, 32] and genetic risk for CHD. To the best of our knowledge, however, no previous research [9, 33, 34] took into account genetic susceptibility in exploring the associations of transport mode with CHD and other common chronic disease outcomes, thereby making it challenging to make a fair comparison. However, we found no strong evidence of multiplicative and additive interaction (except overall and non-commuting transport) between genetic risk and modes of travel for any transport purpose with CHD risk. In general, this is consistent with previous studies showing no strong evidence of interaction between genetic risk and lifestyle-related traits for CHD risk [16, 18, 35, 36]. This observation suggests that using alternatives to the car could benefit the entire population such that individuals at high genetic risk as well as those at low genetic risk would have a lower risk of developing CHD through the use of a more active transport mode. These findings support the current public health guidelines [6] that adults can use more physically active travel options as a way of undertaking daily-life physical activity in the context of transportation. Moreover, our study informs public health interventions customised to individuals at high genetic risk of CHD aiming to prevent or delay the onset of cardiovascular events through lifestyle modification [37, 38]. Such precision medicine approaches [39, 40] have the potential to serve as key cardiovascular disease prevention strategies supplemental to public health policies, and societal and community-based interventions promoting active transport [41].

An advantage of this study is the large number of participants (*n*=333,426) as well as incident CHD cases (*n*=13,730) accrued over a relatively long period of follow-up (a median 13.8 years), which ensured sufficient statistical power for the analyses performed. In addition, we removed the first 2 years of CHD follow-up to reduce the likelihood of reverse causation in the main analysis, with an additional 2 years of follow-up removed in one of the sensitivity analyses. Furthermore, we explored additive interaction as well as multiplicative interaction to better address the potential biological interactions and public health relevance [42, 43].

This study also has some limitations worth noting. First, causal inference is challenging since this is an observational study. Moreover, our analyses only included white British individuals, and, therefore, lack generalisability towards individuals of non-European ancestry as well as those living in other contexts where use of alternatives to the car might be more common. In addition, we did not use information on the duration and/or distance of transport, and hence the present results do not provide quantified exposure information of relevance to dose-response relationships of transport mode with risk of CHD. Furthermore, participants were allowed to choose more than one mode of transport among the four possible options (e.g., cars, walking, cycling and public transport) for both non-commuting and commuting, and a substantially large proportion of individuals reported using more than one mode of transport for non-commuting (e.g., approximately 49%) and commuting (e.g., approximately 20%), thereby resulting in small numbers of CHD incidence cases in the PRS-stratified analyses (Supplemental Table 10). Therefore, it was not feasible to generate and use multiple mutually exclusive categories of transport modes (e.g., car users, active travellers [bike/cycling] and public transport users) in the same analysis. Moreover, recall bias may be present in the assessment of transport modes due to the use of questionnaires to assess this exposure; however, modes of transport for a person are likely to be similar across days and months, so would be easier to recall than more sporadic activities. Similarly, confounding from unmeasured covariates and residual confounding from poorly measured covariates may also be present in our analyses.

## Conclusion

The exclusive use of cars was, in general, predictive of subsequent CHD, irrespective of genetic susceptibility to CHD. All individuals, including those at high genetic risk, using alternatives to the car had a relatively lower risk of developing CHD compared with those using cars exclusively. Encouraging more active patterns of travel should be a key lifestyle behavioural goal for everyone including individuals whose genetic risk of CHD is high, and policy makers across both health and transport sectors should work together to create physical and policy environments that facilitate such healthy choices.

## Supplementary Information

**Additional file 1: Supplemental Table 1.** A list of singlenucleotide polymorphisms (SNP) for risk of coronary heart disease (CHD)(*N*=300). **Supplemental Table 2.** Statistics for multicollinearity for eachcovariate as identified from Cox regression models. **Supplemental Table 3.** Multiple imputation for missing data. **Supplemental Table 4.** Associations of mode oftransport with incident coronary heart disease (CHD) after excluding incidentCHD events accrued over the first 4 years of follow-up. **Supplemental Table 5.** Associations of mode of transport withincident coronary heart disease (CHD) after retaining 1 participant randomly selected from each set ofgenetically related individuals (at 2nd degree). **Supplemental Table 6.** Associations of mode of transport with incident coronary heart disease(CHD) using a weighted polygenic risk score calculated using 46 lead SNPs (from46 loci) which were genome-wide significant (*p*-value: 5×10^-8^) and inlow linkage disequilibrium (*r*2<0.001). **SupplementalTable 7.** Associations of mode of transport with incident coronary heart disease(CHD) using data censored on March 1st, 2020 to take into consideration thepossibility of CHD cases not diagnosed due to participants’ fear of visitinghospitals during COVID-19. **SupplementalTable 8.** Associations of mode of transport with incident coronary heart disease(CHD) using values imputed for the covariates missing, assuming data missing atrandom. **SupplementalTable 9.** Joint associations of mode of transport and genetic risk with incidentcoronary heart disease (CHD). **Supplemental Table 10.** Number of participants and coronary heart disease(CHD) cases by different categories of mode of transport. **Supplemental Figure 1.** Distribution of the calculated polygenic riskscores (PRS) for coronary heart disease using 300 known SNPs. **Supplemental Figure 2.** Quantification of transport mode variables. **Supplemental Figure 3.** An interaction directed acyclic graph (IDAG)describing the conceptual framework for the interplay of active transport andgenetic risk of coronary heart disease (CHD) in relation to risk of CHD. **Supplemental Figure 4.** Distribution of the calculated polygenic riskscore (PRS) for coronary heart disease using 46 lead SNPs (from 46 loci) whichwere genome-wide significant (*p*-value: 5×10^-8^) and in low linkage disequilibrium(*r*^2^<0.001).

## Data Availability

This study used data from the UK Biobank study. Application for access to UK Biobank data can be submitted at the following link: http://www.ukbiobank.ac.uk/register-apply/

## Abbreviations

CHD: Coronary heart disease
CI: Confidence interval
COVID-19: Coronavirus disease 2019
HR: Hazard ratios
ICD: International classification of diseases
PRS: Polygenic risk scores
SNP: Single nucleotide polymorphisms
