# Supplementary material for: Mode of transport, genetic susceptibility, and incidence of coronary heart disease
Source: Int J Behav Nutr Phys Act. 2023 Jul 4;20:79. doi: 10.1186/s12966-023-01484-4 (PMC10320975; doi:10.1186/s12966-023-01484-4)
Supplement: Supplementary file 1 — Additional file 1: Supplemental Table 1. A list of singlenucleotide polymorphisms (SNP) for risk of coronary heart disease (CHD)(N=300). Supplemental Table 2. Statistics for multicollinearity for eachcovariate as identified from Cox regression models. Supplemental Table 3. Multiple imputation for missing data. Supplemental Table 4. Associations of mode oftransport with incident coronary heart disease (CHD) after excluding incidentCHD events accrued over the first 4 years of follow-up. Supplemental Table 5. Associations of mode of transport withincident coronary heart disease (CHD) after retaining 1 participant randomly selected from each set ofgenetically related individuals (at 2nd degree). Supplemental Table 6. Associations of mode of transport with incident coronary heart disease(CHD) using a weighted polygenic risk score calculated using 46 lead SNPs (from46 loci) which were genome-wide significant (p-value: 5×10^-8^) and inlow linkage disequilibrium (r2<0.001). SupplementalTable 7. Associations of mode of transport with incident coronary heart disease(CHD) using data censored on March 1st, 2020 to take into consideration thepossibility of CHD cases not diagnosed due to participants’ fear of visitinghospitals during COVID-19. SupplementalTable 8. Associations of mode of transport with incident coronary heart disease(CHD) using values imputed for the covariates missing, assuming data missing atrandom. SupplementalTable 9. Joint associations of mode of transport and genetic risk with incidentcoronary heart disease (CHD). Supplemental Table 10. Number of participants and coronary heart disease(CHD) cases by different categories of mode of transport. Supplemental Figure 1. Distribution of the calculated polygenic riskscores (PRS) for coronary heart disease using 300 known SNPs. Supplemental Figure 2. Quantification of transport mode variables. Supplemental Figure 3. An interaction directed acyclic graph (IDAG)describing the conceptual framework for the interplay of ac [file 12966_2023_1484_MOESM1_ESM.docx]

# Supplemental Table 1. A list of single nucleotide polymorphisms (SNP) for risk of coronary heart disease (CHD) (N=300).

| **SNPs** | **Effect allele** | **Other allele** | **Beta** | ***p*-value** |
| --- | --- | --- | --- | --- |
| rs2843152 | C | G | 0.042 | 1.34*10^-5^ |
| rs35465346 | G | A | 0.055 | 5.91*10^-6^ |
| rs7538207 | C | T | 0.098 | 5.73*10^-6^ |
| rs11811081 | C | A | 0.077 | 5.65*10^-5^ |
| rs12733730 | A | G | 0.046 | 1.79*10^-5^ |
| rs11485595 | T | C | 0.04 | 4.00*10^-6^ |
| rs34232196 | C | T | 0.055 | 2.87*10^-8^ |
| rs11591147^#^ | G | T | 0.221 | 2.84*10^-10^ |
| rs17111652 | T | C | 0.082 | 1.34*10^-5^ |
| rs6665249 | A | G | 0.042 | 1.54*10^-5^ |
| rs56170783^#^ | A | C | 0.104 | 2.14*10^-12^ |
| rs2149821 | A | T | 0.039 | 3.17*10^-5^ |
| rs10890013 | T | C | 0.035 | 2.26*10^-5^ |
| rs113832197 | T | C | 0.081 | 3.40*10^-5^ |
| rs7528419^#^ | A | G | 0.109 | 3.77*10^-27^ |
| rs11552449 | T | C | 0.045 | 3.90*10^-5^ |
| rs10305649 | A | C | 0.114 | 2.87*10^-6^ |
| rs11810571 | G | C | 0.058 | 2.21*10^-8^ |
| rs6689306^#^ | A | G | 0.05 | 1.46*10^-9^ |
| rs2789422 | G | A | 0.035 | 4.63*10^-5^ |
| rs6413828 | A | T | 0.038 | 1.77*10^-5^ |
| rs183692864 | G | A | 0.244 | 5.49*10^-5^ |
| rs1892094 | C | T | 0.02 | 1.55*10^-2^ |
| rs6700559 | C | T | 0.022 | 6.96*10^-3^ |
| rs2820315 | T | C | 0.043 | 2.09*10^-6^ |
| rs67180937^#^ | G | T | 0.071 | 8.45*10^-14^ |
| rs17464857 | T | G | 0.06 | 6.90*10^-7^ |
| rs3755549 | C | T | 0.034 | 2.64*10^-5^ |
| rs2709437 | T | C | 0.034 | 4.38*10^-5^ |
| rs16986953^#^ | A | G | 0.105 | 4.77*10^-10^ |
| rs585967^#^ | C | A | 0.066 | 2.76*10^-8^ |
| rs58560619 | C | T | 0.036 | 1.65*10^-5^ |
| rs4299376^#^ | G | T | 0.055 | 5.65*10^-10^ |
| rs4076834 | T | G | 0.1 | 1.23*10^-8^ |
| rs139591697 | T | C | 0.102 | 5.15*10^-5^ |
| rs72375964 | A | G | 0.035 | 5.01*10^-5^ |
| rs7568458^#^ | A | T | 0.061 | 2.39*10^-13^ |
| rs149366039 | T | C | 0.731 | 4.03*10^-5^ |
| rs7578433 | T | C | 0.07 | 3.87*10^-5^ |
| rs79716828 | C | A | 0.111 | 8.63*10^-6^ |
| rs6761276 | T | C | 0.036 | 2.66*10^-5^ |
| rs7570006 | C | T | 0.048 | 3.78*10^-5^ |
| rs17678683 | G | T | 0.077 | 1.15*10^-7^ |
| rs35500812 | A | C | 0.042 | 3.20*10^-6^ |
| rs12619842 | G | C | 0.048 | 8.57*10^-6^ |
| rs62172372 | A | G | 0.046 | 3.61*10^-5^ |
| rs114123510^#^ | A | T | 0.118 | 2.88*10^-19^ |
| rs2011559 | G | A | 0.054 | 2.44*10^-5^ |
| rs1250229^#^ | T | C | 0.069 | 1.85*10^-13^ |
| rs2161967 | T | G | 0.039 | 6.21*10^-6^ |
| rs2972146 | T | G | 0.047 | 6.50*10^-8^ |
| rs10168194 | C | G | 0.039 | 6.78*10^-6^ |
| rs13003675 | T | C | 0.042 | 1.72*10^-6^ |
| rs10929113 | C | T | 0.042 | 5.24*10^-5^ |
| rs143803699 | G | C | 0.12 | 2.95*10^-5^ |
| rs748431 | G | T | 0.041 | 9.04*10^-7^ |
| rs3821396 | G | A | 0.06 | 6.57*10^-6^ |
| rs7623687^#^ | A | C | 0.072 | 3.72*10^-9^ |
| rs77622129 | A | G | 0.094 | 2.00*10^-5^ |
| rs62253653 | A | G | 0.038 | 4.35*10^-5^ |
| rs71331765 | G | C | 0.051 | 4.47*10^-5^ |
| rs6787409 | C | T | 0.039 | 1.63*10^-5^ |
| rs9818870^#^ | T | C | 0.068 | 7.82*10^-9^ |
| rs4632520 | C | T | 0.038 | 2.90*10^-5^ |
| rs12493885^#^ | C | G | 0.071 | 3.29*10^-8^ |
| rs10513507 | C | T | 0.036 | 2.20*10^-5^ |
| rs34229028 | G | A | 0.037 | 1.25*10^-5^ |
| rs9869263 | G | A | 0.048 | 3.74*10^-5^ |
| rs113148244 | G | T | 0.144 | 2.20*10^-5^ |
| rs16994919 | A | G | 0.06 | 9.55*10^-6^ |
| rs2616407 | C | T | 0.049 | 5.60*10^-6^ |
| rs13134452 | C | T | 0.037 | 3.16*10^-5^ |
| rs72627509 | G | C | 0.054 | 8.10*10^-8^ |
| rs10857147^#^ | T | A | 0.054 | 8.96*10^-9^ |
| rs138495951 | G | A | 0.16 | 3.84*10^-5^ |
| rs7678555 | C | A | 0.048 | 1.43*10^-7^ |
| rs144059514 | G | A | 0.085 | 4.50*10^-3^ |
| rs13109172 | C | T | 0.038 | 5.73*10^-6^ |
| rs4593108 | C | G | 0.058 | 1.95*10^-8^ |
| rs6841581^#^ | A | G | 0.068 | 4.57*10^-10^ |
| rs7435973 | G | A | 0.059 | 4.51*10^-7^ |
| rs3796587^#^ | C | G | 0.063 | 1.24*10^-9^ |
| rs869396 | C | A | 0.039 | 1.85*10^-6^ |
| rs11728590 | G | T | 0.037 | 1.97*10^-5^ |
| rs71600236 | C | G | 0.039 | 6.54*10^-6^ |
| rs112941079 | A | G | 0.059 | 3.86*10^-6^ |
| rs5868014 | G | A | 0.052 | 1.04*10^-6^ |
| rs111777100 | A | G | 0.093 | 5.35*10^-5^ |
| rs288187 | C | T | 0.046 | 2.63*10^-5^ |
| rs1800449 | T | C | 0.056 | 4.06*10^-7^ |
| rs1500187 | G | A | 0.037 | 9.73*10^-6^ |
| rs6883598 | C | A | 0.039 | 2.32*10^-5^ |
| rs273909 | G | A | 0.053 | 9.94*10^-4^ |
| rs251023 | G | A | 0.038 | 8.34*10^-6^ |
| rs11955380 | C | A | 0.06 | 1.28*10^-5^ |
| rs3776307 | G | A | 0.038 | 9.40*10^-6^ |
| rs6860540 | G | A | 0.035 | 5.49*10^-5^ |
| rs9501744 | C | T | 0.064 | 1.08*10^-6^ |
| rs421329 | C | T | 0.049 | 1.55*10^-6^ |
| rs742115 | C | T | 0.036 | 2.86*10^-5^ |
| rs6458138 | G | A | 0.065 | 6.13*10^-5^ |
| rs9349379^#^ | G | A | 0.105 | 9.95*10^-36^ |
| rs13200993^#^ | T | C | 0.05 | 5.60*10^-9^ |
| rs3130683 | T | C | 0.077 | 2.77*10^-8^ |
| rs9268402 | A | G | 0.014 | 1.28*10^-1^ |
| rs4472337 | T | C | 0.055 | 2.42*10^-6^ |
| rs17609940 | G | C | 0.029 | 7.11*10^-3^ |
| rs56015508 | C | A | 0.054 | 1.08*10^-7^ |
| rs1214752 | C | T | 0.039 | 3.26*10^-6^ |
| rs6905288 | A | G | 0.039 | 3.26*10^-6^ |
| rs1330633 | G | A | 0.067 | 5.89*10^-5^ |
| rs194937 | A | G | 0.048 | 1.26*10^-5^ |
| rs11153071 | G | A | 0.05 | 3.00*10^-6^ |
| rs9398803 | A | G | 0.034 | 6.23*10^-5^ |
| rs12202017^#^ | A | G | 0.066 | 6.02*10^-14^ |
| rs9493752 | A | G | 0.154 | 7.05*10^-6^ |
| rs2492304 | A | T | 0.033 | 5.59*10^-5^ |
| rs2153219 | A | G | 0.051 | 2.59*10^-6^ |
| rs55730499^#^ | T | C | 0.268 | 5.64*10^-49^ |
| rs186696265 | T | C | 0.466 | 8.97*10^-36^ |
| rs4252198 | G | C | 0.152 | 1.66*10^-5^ |
| rs79018195 | C | T | 0.304 | 1.26*10^-6^ |
| rs41269133 | T | C | 0.079 | 4.78*10^-8^ |
| rs9364552 | C | G | 0.035 | 1.50*10^-5^ |
| rs6956990 | C | T | 0.115 | 9.11*10^-6^ |
| rs11509880 | A | G | 0.036 | 2.11*10^-5^ |
| rs2107595^#^ | A | G | 0.074 | 3.41*10^-13^ |
| rs55889159 | A | C | 0.035 | 5.11*10^-5^ |
| rs78850423 | A | G | 0.143 | 9.49*10^-6^ |
| rs2971672 | C | A | 0.036 | 1.90*10^-5^ |
| rs1088868 | G | A | 0.04 | 3.84*10^-5^ |
| rs35146811 | C | A | 0.042 | 5.36*10^-6^ |
| rs112370447 | T | C | 0.045 | 9.62*10^-7^ |
| rs10953541 | C | T | 0.027 | 5.77*10^-3^ |
| rs2024233 | G | A | 0.037 | 2.85*10^-5^ |
| rs11556924^#^ | C | T | 0.067 | 6.26*10^-13^ |
| rs2286198 | G | A | 0.051 | 3.42*10^-7^ |
| rs3918226^#^ | T | C | 0.125 | 1.58*10^-12^ |
| rs2083636 | T | G | 0.051 | 6.44*10^-8^ |
| rs28597716 | A | G | 0.051 | 6.85*10^-6^ |
| rs16885577 | G | A | 0.049 | 2.56*10^-5^ |
| rs10109493 | A | G | 0.061 | 4.93*10^-5^ |
| rs72658939 | G | C | 0.046 | 2.80*10^-5^ |
| rs77211063 | T | C | 0.11 | 2.30*10^-5^ |
| rs10955380 | C | A | 0.04 | 1.19*10^-5^ |
| rs2954029^#^ | A | T | 0.06 | 5.24*10^-13^ |
| rs117938894 | G | A | 0.161 | 3.73*10^-5^ |
| rs75824083 | C | T | 0.907 | 5.09*10^-5^ |
| rs58594043 | A | G | 0.051 | 3.14*10^-5^ |
| rs34914400 | T | C | 0.813 | 1.60*10^-5^ |
| rs2891168^#^ | G | A | 0.173 | 5.23*10^-104^ |
| rs3217992 | T | C | 0.122 | 5.50*10^-49^ |
| rs1333050 | T | C | 0.124 | 2.39*10^-41^ |
| rs4149311 | T | C | 0.052 | 9.06*10^-6^ |
| rs1967604 | A | G | 0.037 | 5.01*10^-5^ |
| rs111245230 | C | T | 0.109 | 8.29*10^-7^ |
| rs781622 | T | C | 0.036 | 2.13*10^-5^ |
| rs77275410 | C | T | 0.061 | 2.55*10^-5^ |
| rs10818583 | A | G | 0.043 | 4.85*10^-6^ |
| rs507666^#^ | A | G | 0.074 | 1.34*10^-12^ |
| rs11257613 | G | A | 0.035 | 1.82*10^-5^ |
| rs7094201 | G | A | 0.062 | 5.77*10^-5^ |
| rs1887318^#^ | T | C | 0.058 | 4.12*10^-12^ |
| rs1870634^#^ | G | T | 0.062 | 5.51*10^-13^ |
| rs1657345 | A | G | 0.081 | 4.58*10^-12^ |
| rs17726488 | T | C | 0.103 | 1.01*10^-5^ |
| rs4691 | T | C | 0.041 | 8.67*10^-6^ |
| rs7098414 | A | C | 0.046 | 2.66*10^-6^ |
| rs2246942^#^ | G | A | 0.076 | 3.51*10^-16^ |
| rs59898454 | A | G | 0.147 | 2.09*10^-6^ |
| rs11191416^#^ | T | G | 0.073 | 5.58*10^-9^ |
| rs12252333 | G | A | 0.047 | 4.90*10^-6^ |
| rs2257129 | C | T | 0.096 | 1.64*10^-6^ |
| rs2281674 | C | G | 0.064 | 4.68*10^-5^ |
| rs28596486 | C | T | 0.052 | 2.91*10^-6^ |
| rs56210063 | C | G | 0.068 | 4.00*10^-5^ |
| rs10840293^#^ | A | G | 0.049 | 6.88*10^-9^ |
| rs11042937 | T | G | 0.011 | 2.02*10^-1^ |
| rs3993105 | T | C | 0.047 | 1.06*10^-7^ |
| rs11462682 | G | A | 0.044 | 8.35*10^-5^ |
| rs146039567 | C | A | 0.149 | 2.20*10^-5^ |
| rs2306029 | T | C | 0.039 | 1.57*10^-5^ |
| rs2727020 | C | G | 0.042 | 4.70*10^-6^ |
| rs12146487 | G | A | 0.048 | 2.28*10^-5^ |
| rs12801636 | G | A | 0.043 | 7.75*10^-6^ |
| rs571353 | C | T | 0.043 | 3.47*10^-6^ |
| rs634552 | G | T | 0.05 | 4.71*10^-5^ |
| rs3133293 | G | T | 0.041 | 3.34*10^-6^ |
| rs17712139 | G | A | 0.041 | 3.62*10^-5^ |
| rs2212437 | A | G | 0.04 | 7.63*10^-6^ |
| rs2839812^#^ | T | A | 0.06 | 1.99*10^-11^ |
| rs567040 | C | T | 0.037 | 6.21*10^-5^ |
| rs964184 | G | C | 0.051 | 4.68*10^-6^ |
| rs3782774 | G | A | 0.036 | 2.17*10^-5^ |
| rs3861086 | C | T | 0.045 | 6.34*10^-7^ |
| rs11170820 | G | C | 0.089 | 2.38*10^-7^ |
| rs56245751 | T | C | 0.061 | 1.13*10^-5^ |
| rs11172113 | C | T | 0.036 | 2.44*10^-5^ |
| rs2229357 | G | A | 0.047 | 3.39*10^-6^ |
| rs6538176 | T | C | 0.047 | 6.24*10^-6^ |
| rs11115214 | C | T | 0.042 | 3.71*10^-5^ |
| rs2681472^#^ | G | A | 0.066 | 7.63*10^-11^ |
| rs10774625^#^ | A | G | 0.064 | 9.22*10^-14^ |
| rs2244608^#^ | G | A | 0.051 | 2.32*10^-9^ |
| rs11057401 | T | A | 0.044 | 1.32*10^-6^ |
| rs11057830 | A | G | 0.069 | 4.24*10^-9^ |
| rs1924981 | T | C | 0.046 | 1.86*10^-7^ |
| rs9591012 | G | A | 0.038 | 1.87*10^-5^ |
| rs73468973 | A | G | 0.043 | 5.68*10^-5^ |
| rs75535189 | C | T | 0.262 | 1.01*10^-5^ |
| rs9515203^#^ | T | C | 0.062 | 6.48*10^-10^ |
| rs4773141 | G | C | 0.059 | 9.46*10^-10^ |
| rs9588107 | A | G | 0.033 | 7.80*10^-5^ |
| rs12867664 | A | G | 0.086 | 1.57*10^-5^ |
| rs17102313 | T | C | 0.615 | 4.64*10^-5^ |
| rs12891473 | C | T | 0.035 | 2.33*10^-5^ |
| rs4506804 | T | G | 0.034 | 3.14*10^-5^ |
| rs3832966 | C | T | 0.037 | 4.67*10^-6^ |
| rs112635299 | G | T | 0.163 | 1.65*10^-5^ |
| rs10139550^#^ | G | C | 0.051 | 1.84*10^-9^ |
| rs113025579 | C | T | 0.125 | 4.94*10^-5^ |
| rs147580454 | C | T | 0.033 | 9.72*10^-5^ |
| rs6494488 | A | G | 0.034 | 1.80*10^-3^ |
| rs72743461^#^ | C | A | 0.071 | 4.81*10^-12^ |
| rs7164479^#^ | T | C | 0.072 | 6.38*10^-18^ |
| rs2083460 | T | C | 0.072 | 1.41*10^-7^ |
| rs2071382^#^ | T | C | 0.062 | 7.14*10^-13^ |
| rs17581137 | A | C | 0.042 | 1.38*10^-5^ |
| rs116082507 | T | C | 1.204 | 4.13*10^-6^ |
| rs7185993 | T | C | 0.036 | 1.23*10^-5^ |
| rs247616 | C | T | 0.044 | 1.01*10^-6^ |
| rs35259348 | C | G | 0.051 | 1.15*10^-7^ |
| rs1050362 | A | C | 0.029 | 4.84*10^-4^ |
| rs9929108 | T | G | 0.047 | 2.32*10^-7^ |
| rs3851738 | C | G | 0.041 | 6.67*10^-7^ |
| rs7500448^#^ | A | G | 0.059 | 5.14*10^-9^ |
| rs1968266 | T | C | 0.037 | 5.23*10^-5^ |
| rs117592425 | A | C | 0.203 | 4.14*10^-6^ |
| rs113348108 | G | A | 0.044 | 2.02*10^-7^ |
| rs8068571 | T | C | 0.042 | 1.76*10^-5^ |
| rs9897596 | T | C | 0.039 | 3.13*10^-6^ |
| rs13723 | G | A | 0.035 | 2.39*10^-5^ |
| rs148720362 | C | T | 0.035 | 5.06*10^-4^ |
| rs1122326 | C | A | 0.05 | 2.76*10^-6^ |
| rs8068844 | C | T | 0.043 | 3.93*10^-7^ |
| rs17608766 | C | T | 0.044 | 6.07*10^-4^ |
| rs46522 | T | C | 0.033 | 9.31*10^-5^ |
| rs4643373 | T | C | 0.046 | 1.20*10^-6^ |
| rs62076439 | T | G | 0.044 | 7.83*10^-7^ |
| rs8068952^#^ | G | C | 0.07 | 1.41*10^-9^ |
| rs7212798 | C | T | 0.063 | 4.37*10^-8^ |
| rs6504218 | G | A | 0.041 | 9.41*10^-7^ |
| rs11077501 | C | T | 0.037 | 2.19*10^-5^ |
| rs75589791 | G | A | 0.065 | 4.58*10^-5^ |
| rs35489971 | A | G | 0.054 | 1.06*10^-6^ |
| rs11654510 | C | A | 0.058 | 9.46*10^-6^ |
| rs7211674 | C | A | 0.034 | 6.14*10^-5^ |
| rs9951447 | C | T | 0.038 | 4.37*10^-6^ |
| rs178002 | G | A | 0.042 | 1.91*10^-6^ |
| rs12922 | A | C | 0.05 | 2.92*10^-5^ |
| rs833509 | C | T | 0.039 | 2.58*10^-5^ |
| rs948937 | A | T | 0.034 | 6.24*10^-5^ |
| rs35614134 | A | C | 0.039 | 2.30*10^-5^ |
| rs663129 | A | G | 0.04 | 1.82*10^-5^ |
| rs116843064 | G | A | 0.159 | 2.87*10^-7^ |
| rs111397563 | T | C | 0.052 | 1.28*10^-8^ |
| rs6511720^#^ | G | T | 0.128 | 7.88*10^-22^ |
| rs2738448 | G | C | 0.034 | 5.04*10^-5^ |
| rs167479 | G | T | 0.04 | 2.26*10^-6^ |
| rs73015715 | T | C | 0.049 | 2.32*10^-6^ |
| rs78030362 | G | A | 0.069 | 5.46*10^-5^ |
| rs10423964 | T | C | 0.039 | 2.38*10^-5^ |
| rs10417115 | C | T | 0.068 | 2.25*10^-5^ |
| rs34322801 | C | G | 0.05 | 6.03*10^-6^ |
| rs73045269 | T | C | 0.064 | 1.71*10^-7^ |
| rs4760 | G | A | 0.054 | 2.56*10^-5^ |
| rs7412^#^ | C | T | 0.143 | 2.17*10^-19^ |
| rs56131196 | A | G | 0.082 | 2.71*10^-12^ |
| rs1964272 | G | A | 0.044 | 2.29*10^-7^ |
| rs425105 | C | T | 0.047 | 4.72*10^-5^ |
| rs13734 | A | G | 0.043 | 2.03*10^-5^ |
| rs59909520 | C | T | 0.059 | 1.04*10^-5^ |
| rs867186 | A | G | 0.057 | 1.47*10^-5^ |
| rs117113213 | A | G | 0.131 | 1.19*10^-6^ |
| rs6129767 | G | T | 0.04 | 1.04*10^-5^ |
| rs56313611 | C | T | 0.058 | 1.39*10^-6^ |
| rs259983 | C | A | 0.056 | 2.89*10^-6^ |
| rs3813452 | T | C | 0.035 | 3.67*10^-5^ |
| rs2832275 | T | A | 0.051 | 2.04*10^-6^ |
| rs75187018 | G | A | 0.136 | 1.85*10^-5^ |
| rs28451064^#^ | A | G | 0.133 | 2.62*10^-23^ |
| rs743339 | C | T | 0.075 | 3.05*10^-14^ |
| rs117696200 | T | G | 0.079 | 1.82*10^-5^ |
| rs2836621 | T | C | 0.033 | 4.68*10^-5^ |
| rs35219138 | C | A | 0.034 | 4.83*10^-5^ |
| rs9604969 | A | G | 0.06 | 5.69*10^-5^ |
| rs71313931 | G | C | 0.039 | 1.87*10^-5^ |
| rs11287675 | C | T | 0.035 | 2.78*10^-5^ |
| rs12485143 | C | T | 0.067 | 2.72*10^-5^ |
| rs468224 | A | G | 0.043 | 8.85*10^-6^ |

Note: “#” specifies 46 lead SNPs (from 46 loci) which were genome-wide significant (*p*-value: 5×10^-8^) and in low linkage disequilibrium (r^2^<0.001).

# Supplemental Table 2. Statistics for multicollinearity for each covariate as identified from Cox regression models.

| Covariant | Overall transport model^a^ | | |  | Non-commuting transport model^b^ | | |  | Commuting transport model^c^ | | |
| --- | --- | --- | --- | --- | --- | --- | --- | --- | --- | --- | --- |
|  | GVIF | df | ${GVIF}^{1/2df}$ |  | GVIF | df | ${GVIF}^{1/2df}$ |  | GVIF | df | ${GVIF}^{1/2df}$ |
| Body mass index | 1.19 | 1 | 1.09 |  | 1.20 | 1 | 1.10 |  | 1.19 | 1 | 1.09 |
| Sex | 1.16 | 1 | 1.08 |  | 1.21 | 1 | 1.10 |  | 1.16 | 1 | 1.08 |
| Townsend Deprivation Index | 1.13 | 1 | 1.06 |  | 1.12 | 1 | 1.06 |  | 1.12 | 1 | 1.06 |
| Smoking | 1.16 | 2 | 1.04 |  | 1.16 | 2 | 1.04 |  | 1.16 | 2 | 1.06 |
| Alcohol intake | 1.14 | 3 | 1.02 |  | 1.16 | 3 | 1.02 |  | 1.14 | 3 | 1.02 |
| Salt intake | 1.10 | 3 | 1.02 |  | 1.10 | 3 | 1.02 |  | 1.10 | 3 | 1.02 |
| Oily fish consumption | 1.14 | 3 | 1.02 |  | 1.12 | 3 | 1.02 |  | 1.14 | 3 | 1.02 |
| Coffee intake | 1.04 | 1 | 1.02 |  | 1.03 | 1 | 1.02 |  | 1.04 | 1 | 1.02 |
| Fruit and vegetable intake | 1.18 | 4 | 1.02 |  | 1.15 | 4 | 1.02 |  | 1.18 | 4 | 1.02 |
| Processed/red meat intake | 1.14 | 1 | 1.07 |  | 1.12 | 1 | 1.06 |  | 1.14 | 1 | 1.06 |
| Blood-pressure-lowering medication use | 1.35 | 1 | 1.16 |  | 1.32 | 1 | 1.15 |  | 1.35 | 1 | 1.16 |
| Cholesterol-lowering medication use | 1.30 | 1 | 1.14 |  | 1.28 | 1 | 1.13 |  | 1.30 | 1 | 1.14 |
| TV viewing | 1.11 | 1 | 1.05 |  | 1.14 | 1 | 1.07 |  | 1.11 | 1 | 1.05 |
| Computer use | 1.02 | 1 | 1.01 |  | 1.04 | 1 | 1.02 |  | 1.02 | 1 | 1.01 |
| Sleep | 1.02 | 1 | 1.01 |  | 1.01 | 1 | 1.01 |  | 1.02 | 1 | 1.01 |
| Walking for pleasure | 1.04 | 1 | 1.02 |  | 1.05 | 1 | 1.03 |  | 1.03 | 1 | 1.01 |
| Light do-it-yourself activities | 1.11 | 1 | 1.05 |  | 1.08 | 1 | 1.04 |  | 1.11 | 1 | 1.05 |
| Heavy do-it-yourself activities | 1.12 | 1 | 1.06 |  | 1.10 | 1 | 1.04 |  | 1.12 | 1 | 1.06 |
| Strenuous sports | 1.15 | 1 | 1.07 |  | 1.08 | 1 | 1.04 |  | 1.15 | 1 | 1.07 |
| Other exercises | 1.17 | 1 | 1.08 |  | 1.09 | 1 | 1.04 |  | 1.17 | 1 | 1.08 |

Note: a. Model used the categories of overall transport mode as the exposure; b. Model used the categories of non-commuting transport mode as the exposure; c. Model used the categories of commuting transport mode as the exposure. Abbreviation: GIF- generalized variance inflation factor; df: degree of freedom.

# Supplemental Table 3. Multiple imputation for missing data

| Variables | Complete | Incomplete | Imputed | Total |
| --- | --- | --- | --- | --- |
| Age, years | 370,378 | 0 | 0 | 370,378 |
| Sex, n (%) | 370,378 | 0 | 0 | 370,378 |
| Body mass index, kg/m^2^ | 369,525 | 853 | 853 | 370,378 |
| Smoking status, % | 369,206 | 1,172 | 1,172 | 370,378 |
| Townsend Deprivation Index | 369,969 | 409 | 409 | 370,378 |
| Alcohol Consumption Status | 370,105 | 273 | 273 | 370,378 |
| Salt-adding behaviour | 370,368 | 10 | 10 | 370,378 |
| Oily fish consumption | 368,772 | 1,606 | 1,606 | 370,378 |
| Coffee intake (cups per day) | 369,831 | 547 | 547 | 370,378 |
| Fruit and vegetable intake (score ranging from 0-4 based on fresh/dried fruit intake and raw/cooked vegetable intake) | 370,115 | 263 | 263 | 370,378 |
| Red meat intake, days/week (average) | 370,324 | 54 | 54 | 370,378 |
| Hypertension medication use, % | 370,244 | 134 | 134 | 370,378 |
| Cholesterol-lowering medication use, % | 370,244 | 134 | 134 | 370,378 |
| TV-viewing, hours/day | 367,879 | 2,499 | 2,499 | 370,378 |
| Computer use, hours/day | 368,084 | 2,294 | 2,294 | 370,378 |
| Sleep, hours/day | 368,523 | 1,855 | 1,855 | 370,378 |
| total walk for pleasure (minutes per day) | 348,744 | 21,634 | 21,634 | 370,378 |
| total light DIY (minutes per day) | 348,744 | 21,634 | 21,634 | 370,378 |
| total heavy DIY (minutes per day) | 348,744 | 21,634 | 21,634 | 370,378 |
| total strenuous sports (minutes per day) | 348,744 | 21,634 | 21,634 | 370,378 |
| total other exercises (minutes per day) | 348,744 | 21,634 | 21,634 | 370,378 |
| Polygenic risk scores for CHD | 370,378 | 0 | 0 | 370,378 |

# Supplemental Table 4. Associations of mode of transport with incident coronary heart disease (CHD) after excluding incident CHD events accrued over the first 4 years of follow-up.

| Comparison | Number of participants | Number of cases | Crude incident rate per 100,000-person years | Hazard ratio (95% confidence interval) |
| --- | --- | --- | --- | --- |
| **Overall transport (N=176,924)** | | | | |
| Alternatives to the car (Reference) | 48,289 | 1,126 | 169.7 | 1.00 (Reference) |
| Mixed transport mode | 62,776 | 1,774 | 205.2 | 1.11 (1.03, 1.20) |
| Exclusive use of cars | 65,859 | 2,109 | 232.6 | 1.14 (1.06, 1.23) |
| **Non-commuting transport (N=338,420)** | | | |  |
| Alternatives to the car (Reference) | 206,685 | 7,478 | 264.8 | 1.00 (Reference) |
| Exclusive use of cars | 131,735 | 5,084 | 281.7 | 1.09 (1.05, 1.13) |
| **Commuting transport (N=176,924)** | | | |  |
| Alternatives to the car (Reference) | 61,338 | 1,420 | 168.4 | 1.00 (Reference) |
| Exclusive use of cars | 115,586 | 3,589 | 225.6 | 1.15 (1.08, 1.22) |

Note: models were adjusted for age (underlying timescale), sex, body mass index, smoking (never, previous, current), alcohol intake (never, previous, currently <3 times/week, currently ≥3 times/week), salt intake (never/rarely, sometimes, usually, always), oily fish intake (never, <once per week, once per week, >once per week), coffee intake (cups per day), fruit and vegetable intake (a composite score based on fresh/dried fruit intake and raw/cooked vegetable intake), processed/red meat intake (days per week), Townsend Deprivation Index (an indicator of area-based socioeconomic status), sleep (≤5, 6, 7, 8 and ≥9hours per day), total leisure-time physical activity (minutes per day; based on walking, non-walking moderate physical activity and non-walking vigorous physical activity), blood-pressure-lowering medication use, cholesterol-lowering medication use, polygenic risk scores, genotyping array type and the first ten principal components of genetic ancestry.

# Supplemental Table 5. Associations of mode of transport with incident coronary heart disease (CHD) after retaining 1 participant randomly selected from each set of genetically related individuals (at 2nd degree).

| Comparison | Number of participants | Number of cases | Crude incident rate per 100,000-person years | Hazard ratio (95% confidence interval) |
| --- | --- | --- | --- | --- |
| **Overall transport (N=165,265)** | | | | |
| Alternatives to the car (Reference) | 45,192 | 1,155 | 186.4 | 1.00 (Reference) |
| Mixed transport mode | 58,505 | 1,759 | 218.7 | 1.08 (0.99, 1.16) |
| Exclusive use of cars | 61,568 | 2,192 | 259.4 | 1.17 (1.08, 1.26) |
| **Non-commuting transport (N=316,303)** | | | |  |
| Alternatives to the car (Reference) | 193,119 | 7,626 | 289.9 | 1.00 (Reference) |
| Exclusive use of cars | 123,184 | 5,189 | 308.5 | 1.08 (1.04, 1.12) |
| **Commuting transport (N=162,256)** | | | |  |
| Alternatives to the car (Reference) | 57,334 | 1,448 | 184.1 | 1.00 (Reference) |
| Exclusive use of cars | 107,931 | 3,658 | 246.8 | 1.15 (1.08, 1.23) |

Note: models were adjusted for age (underlying timescale), sex, body mass index, smoking (never, previous, current), alcohol intake (never, previous, currently <3 times/week, currently ≥3 times/week), salt intake (never/rarely, sometimes, usually, always), oily fish intake (never, <once per week, once per week, >once per week), coffee intake (cups per day), fruit and vegetable intake (a composite score based on fresh/dried fruit intake and raw/cooked vegetable intake), processed/red meat intake (days per week), Townsend Deprivation Index (an indicator of area-based socioeconomic status), sleep (≤5, 6, 7, 8 and ≥9hours per day), total leisure-time physical activity (minutes per day; based on walking, non-walking moderate physical activity and non-walking vigorous physical activity), blood-pressure-lowering medication use, cholesterol-lowering medication use, polygenic risk scores, genotyping array type and the first ten principal components of genetic ancestry.

# Supplemental Table 6. Associations of mode of transport with incident coronary heart disease (CHD) using a weighted polygenic risk score calculated using 46 lead SNPs (from 46 loci) which were genome-wide significant (p-value: 5×10^-8^) and in low linkage disequilibrium (r2<0.001).

| Comparison | Number of participants | Number of cases | Crude incident rate per 100,000-person years | Hazard ratio (95% confidence interval) |
| --- | --- | --- | --- | --- |
| **Overall transport (N=177,370)^a^** | | | | |
| Alternatives to the car (Reference) | 48,381 | 1,218 | 183.5 | 1.00 (Reference) |
| Mixed transport mode | 62,917 | 1,915 | 221.4 | 1.11 (1.03, 1.19) |
| Exclusive use of cars | 66,072 | 2,322 | 255.9 | 1.16 (1.08, 1.25) |
| **Non-commuting transport (N=339,588) ^a^** | | | |  |
| Alternatives to the car (Reference) | 207,377 | 8,170 | 289.1 | 1.00 (Reference) |
| Exclusive use of cars | 132,211 | 5,560 | 307.9 | 1.08 (1.05, 1.12) |
| **Commuting transport (N=177,370) ^a^** | | | |  |
| Alternatives to the car (Reference) | 61,455 | 1,537 | 182.2 | 1.00 (Reference) |
| Exclusive use of cars | 115,915 | 3,918 | 246.1 | 1.16 (1.09, 1.23) |
| **Tertiles of genetic risk (N=339,588) ^b^** | | | | |
| Low (Reference) | 113,133 | 3,502 | 226.0 | 1.00 (Reference) |
| Medium | 113,218 | 4,373 | 283.0 | 1.26 (1.21, 1.32) |
| High | 113,237 | 5,855 | 381.0 | 1.74 (1.67, 1.81) |

Note: a. models were adjusted for age (underlying timescale), sex, body mass index, smoking (never, previous, current), alcohol intake (never, previous, currently <3 times/week, currently ≥3 times/week), salt intake (never/rarely, sometimes, usually, always), oily fish intake (never, <once per week, once per week, >once per week), coffee intake (cups per day), fruit and vegetable intake (a composite score based on fresh/dried fruit intake and raw/cooked vegetable intake), processed/red meat intake (days per week), Townsend Deprivation Index (an indicator of area-based socioeconomic status), sleep (≤5, 6, 7, 8 and ≥9hours per day), total leisure-time physical activity (minutes per day; based on walking, non-walking moderate physical activity and non-walking vigorous physical activity), blood-pressure-lowering medication use, cholesterol-lowering medication use, polygenic risk scores, genotyping array type and the first ten principal components of genetic ancestry.

b. Model was adjusted for age (underlying timescale), sex, genotyping array type and the first ten principal components of genetic ancestry.

# Supplemental Table 7. Associations of mode of transport with incident coronary heart disease (CHD) using data censored on March 1st, 2020 to take into consideration the possibility of CHD cases not diagnosed due to participants’ fear of visiting hospitals during COVID-19.

| Comparison | Number of participants | Number of cases | Crude incident rate per 100,000-person years | Hazard ratio (95% confidence interval) |
| --- | --- | --- | --- | --- |
| **Overall transport (N=177,370) ^a^** | | | | |
| Alternatives to the car (Reference) | 48,381 | 919 | 172.2 | 1.00 (Reference) |
| Mixed transport mode | 62,917 | 1,390 | 199.6 | 1.06 (0.97, 1.15) |
| Exclusive use of cars | 66,072 | 1,756 | 240.2 | 1.15 (1.06, 1.23) |
| **Non-commuting transport (N=339,588) ^a^** | | | |  |
| Alternatives to the car (Reference) | 207,377 | 6,153 | 270.4 | 1.00 (Reference) |
| Exclusive use of cars | 132,211 | 4,249 | 291.9 | 1.10 (1.06, 1.15) |
| **Commuting transport (N=177,370) ^a^** | | | |  |
| Alternatives to the car (Reference) | 61,455 | 1,159 | 170.9 | 1.00 (Reference) |
| Exclusive use of cars | 115,915 | 2,906 | 226.6 | 1.13 (1.05, 1.21) |
| **Tertiles of genetic risk (N=339,588) ^b^** | | | | |
| Low (Reference) | 113,105 | 2,273 | 182.2 | 1.00 (Reference) |
| Medium | 113,222 | 3,393 | 272.6 | 1.52 (1.44, 1.61) |
| High | 113,261 | 4,736 | 382.3 | 2.20 (2.09, 2.31) |

Note: a. models were adjusted for age (underlying timescale), sex, body mass index, smoking (never, previous, current), alcohol intake (never, previous, currently <3 times/week, currently ≥3 times/week), salt intake (never/rarely, sometimes, usually, always), oily fish intake (never, <once per week, once per week, >once per week), coffee intake (cups per day), fruit and vegetable intake (a composite score based on fresh/dried fruit intake and raw/cooked vegetable intake), processed/red meat intake (days per week), Townsend Deprivation Index (an indicator of area-based socioeconomic status), sleep (≤5, 6, 7, 8 and ≥9hours per day), total leisure-time physical activity (minutes per day; based on walking, non-walking moderate physical activity and non-walking vigorous physical activity), blood-pressure-lowering medication use, cholesterol-lowering medication use, polygenic risk scores, genotyping array type and the first ten principal components of genetic ancestry.

b. Model was adjusted for age (underlying timescale), sex, genotyping array type and the first ten principal components of genetic ancestry.

# Supplemental Table 8. Associations of mode of transport with incident coronary heart disease (CHD) using values imputed for the covariates missing, assuming data missing at random.

| Comparison | Number of participants | Number of cases | Crude incident rate per 100,000-person years | Hazard ratio (95% confidence interval) |
| --- | --- | --- | --- | --- |
| **Overall transport (N=191,520)** | | | | |
| Alternatives to the car (Reference) | 52,464 | 1,371 | 190.5 | 1.00 (Reference) |
| Mixed transport mode | 66,552 | 2,075 | 226.9 | 1.11 (1.03, 1.19) |
| Exclusive use of cars | 72,504 | 2,613 | 262.4 | 1.16 (1.08, 1.24) |
| **Non-commuting transport (N=370,384) ^a^** | | | |  |
| Alternatives to the car (Reference) | 223,376 | 9,109 | 299.3 | 1.00 (Reference) |
| Exclusive use of cars | 147,008 | 6,413 | 319.4 | 1.08 (1.04, 1.11) |
| **Commuting transport (N=191,520) ^a^** | | | |  |
| Alternatives to the car (Reference) | 66,783 | 1,728 | 188.5 | 1.00 (Reference) |
| Exclusive use of cars | 124,737 | 4,331 | 252.8 | 1.17 (1.09, 1.23) |
| **Tertiles of genetic risk (N=370, 383) ^b^** | | | | |
| Low (Reference) | 123,374 | 3,556 | 210.2 | 1.00 (Reference) |
| Medium | 123,488 | 5,050 | 299.7 | 1.45 (1.39, 1.51) |
| High | 123,522 | 6,916 | 413.2 | 2.05 (1.97, 2.14) |

Note: a. models were adjusted for age (underlying timescale), sex, body mass index, smoking (never, previous, current), alcohol intake (never, previous, currently <3 times/week, currently ≥3 times/week), salt intake (never/rarely, sometimes, usually, always), oily fish intake (never, <once per week, once per week, >once per week), coffee intake (cups per day), fruit and vegetable intake (a composite score based on fresh/dried fruit intake and raw/cooked vegetable intake), processed/red meat intake (days per week), Townsend Deprivation Index (an indicator of area-based socioeconomic status), sleep (≤5, 6, 7, 8 and ≥9hours per day), total leisure-time physical activity (minutes per day; based on walking, non-walking moderate physical activity and non-walking vigorous physical activity), blood-pressure-lowering medication use, cholesterol-lowering medication use, polygenic risk scores, genotyping array type and the first ten principal components of genetic ancestry.

b. Model was adjusted for age (underlying timescale), sex, genotyping array type and the first ten principal components of genetic ancestry.

# Supplemental Table 9. Joint associations of mode of transport and genetic risk with incident coronary heart disease (CHD).

| Comparison | | Genetic risk | Number of participants | Number of cases | Crude incident rate per 100,000-person years | Hazard ratio (95% confidence interval) |
| --- | --- | --- | --- | --- | --- | --- |
| Overall transport  (N=**177,370**) | Alternatives to the car  (N=48,381) | Low | 15,932 | 195 | 96.5 | 1.00 (Reference) |
|  |  | Medium | 16,149 | 289 | 141.2 | 1.53 (1.30, 1.80) |
|  |  | High | 16,300 | 465 | 226.0 | 2.50 (2.15, 2.90) |
|  | Mixed transport mode  (N=62,917) | Low | 20,681 | 325 | 123.6 | 1.18 (1.01, 1.39) |
|  |  | Medium | 21,053 | 451 | 168.7 | 1.70 (1.46, 1.97) |
|  |  | High | 21,183 | 697 | 259.8 | 2.70 (2.34, 3.12) |
|  | Exclusive use of cars  (N=66,072) | Low | 21,818 | 394 | 141.8 | 1.21 (1.03, 1.41) |
|  |  | Medium | 21,902 | 579 | 208.3 | 1.84 (1.58, 2.13) |
|  |  | High | 22,352 | 821 | 290.4 | 2.82 (2.45, 3.25) |
| Non-commuting transport  (N=**339,588**) | Alternatives to the car  (N=207,377) | Low | 68,945 | 1550 | 178.0 | 1.00 (Reference) |
|  |  | Medium | 69,160 | 2073 | 237.7 | 1.44 (1.36, 1.53) |
|  |  | High | 69,272 | 2820 | 324.1 | 2.02 (1.91, 2.13) |
|  | Exclusive use of cars  (N=132,211) | Low | 44,160 | 1038 | 185.3 | 1.06 (0.98, 1.14) |
|  |  | Medium | 44,062 | 1438 | 258.1 | 1.56 (1.46, 1.67) |
|  |  | High | 43,989 | 1884 | 340.2 | 2.21 (2.08, 2.35) |
| Commuting transport  (N=**177,370**) | Alternatives to the car  (N=61,455) | Low | 20,268 | 247 | 96.0 | 1.00 (Reference) |
|  |  | Medium | 20,533 | 358 | 137.5 | 1.49 (1.29, 1.72) |
|  |  | High | 20,654 | 586 | 224.6 | 2.47 (2.17, 2.82) |
|  | Exclusive use of cars  (N=115,915) | Low | 38,163 | 667 | 137.4 | 1.20 (1.05, 2.03) |
|  |  | Medium | 38,571 | 961 | 196.3 | 1.79 (1.58, 2.03) |
|  |  | High | 39,181 | 1397 | 281.7 | 2.76 (2.44, 3.11) |

Note: Hazard ratios of coronary heart disease along with the corresponding 95% confidence intervals were presented. Models were adjusted for age (underlying timescale), sex, body mass index, smoking (never, previous, current), alcohol intake (never, previous, currently <3 times/week, currently ≥3 times/week), salt intake (never/rarely, sometimes, usually, always), oily fish intake (never, <once per week, once per week, >once per week), coffee intake (cups per day), fruit and vegetable intake (a composite score based on fresh/dried fruit intake and raw/cooked vegetable intake), processed/red meat intake (days per week), Townsend Deprivation Index (an indicator of area-based socioeconomic status), sleep (≤5, 6, 7, 8 and ≥9hours per day), total leisure-time physical activity (minutes per day; based on walking, non-walking moderate physical activity and non-walking vigorous physical activity), blood-pressure-lowering medication use, cholesterol-lowering medication use, polygenic risk scores, genotyping array type and the first ten principal components of genetic ancestry.

# Supplemental Table 10. Number of participants and coronary heart disease (CHD) cases by different categories of mode of transport.

|  | Overall | |  | Low genetic risk | |  | Medium genetic risk | |  | High genetic risk | |
| --- | --- | --- | --- | --- | --- | --- | --- | --- | --- | --- | --- |
|  | Number of participants | Number of CHD cases |  | Number of participants | Number of CHD cases |  | Number of participants | Number of CHD cases |  | Number of participants | Number of CHD cases |
| Commuting transport  (N=177,370) |  |  |  |  |  |  |  |  |  |  |  |
| More than 1 mode of transport | 36,188 | 903 |  | 11,880 | 193 |  | 12,220 | 267 |  | 12,008 | 443 |
| Car/motor vehicle only | 115,915 | 3,918 |  | 37,758 | 824 |  | 38,571 | 1,234 |  | 39,180 | 1,860 |
| Walking only | 8,926 | 203 |  | 2,986 | 36 |  | 2,903 | 67 |  | 3,019 | 100 |
| Public transport only | 12,138 | 337 |  | 3,893 | 59 |  | 4,078 | 105 |  | 4,126 | 173 |
| Cycling only | 4,200 | 94 |  | 1,439 | 21 |  | 1,332 | 23 |  | 1,421 | 50 |
| Non-commuting transport  (N=339,588) |  |  |  |  |  |  |  |  |  |  |  |
| More than 1 mode of transport | 166,082 | 6,465 |  | 55,470 | 1,449 |  | 55,342 | 2,118 |  | 55,270 | 2,898 |
| Car/motor vehicle only | 132,211 | 5,560 |  | 44,160 | 1,255 |  | 44,062 | 1,824 |  | 43,989 | 2,481 |
| Walking only | 22,407 | 816 |  | 7,366 | 179 |  | 7,512 | 260 |  | 7,529 | 377 |
| Public transport only | 15,440 | 772 |  | 4,958 | 211 |  | 5,157 | 238 |  | 5,325 | 323 |
| Cycling only | 3,448 | 117 |  | 1,151 | 24 |  | 1,149 | 38 |  | 1,148 | 55 |


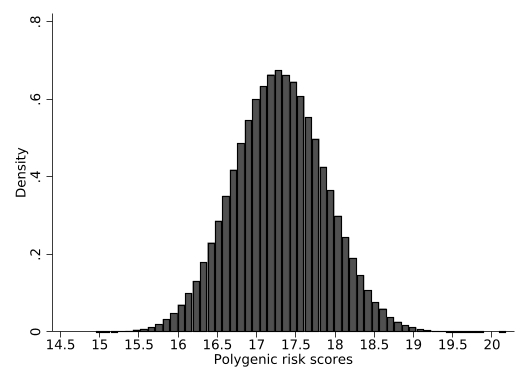


# Supplemental Figure 1. Distribution of the calculated polygenic risk scores (PRS) for coronary heart disease using 300 known SNPs.


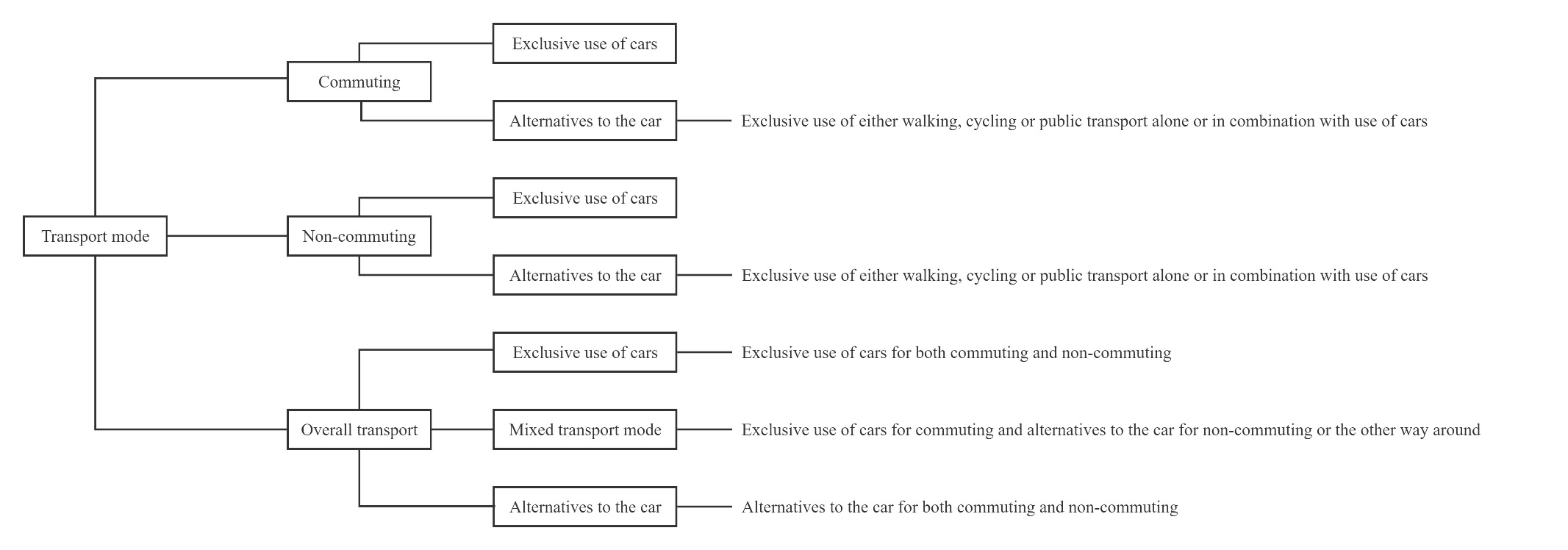


# Supplemental Figure 2. Quantification of transport mode variables


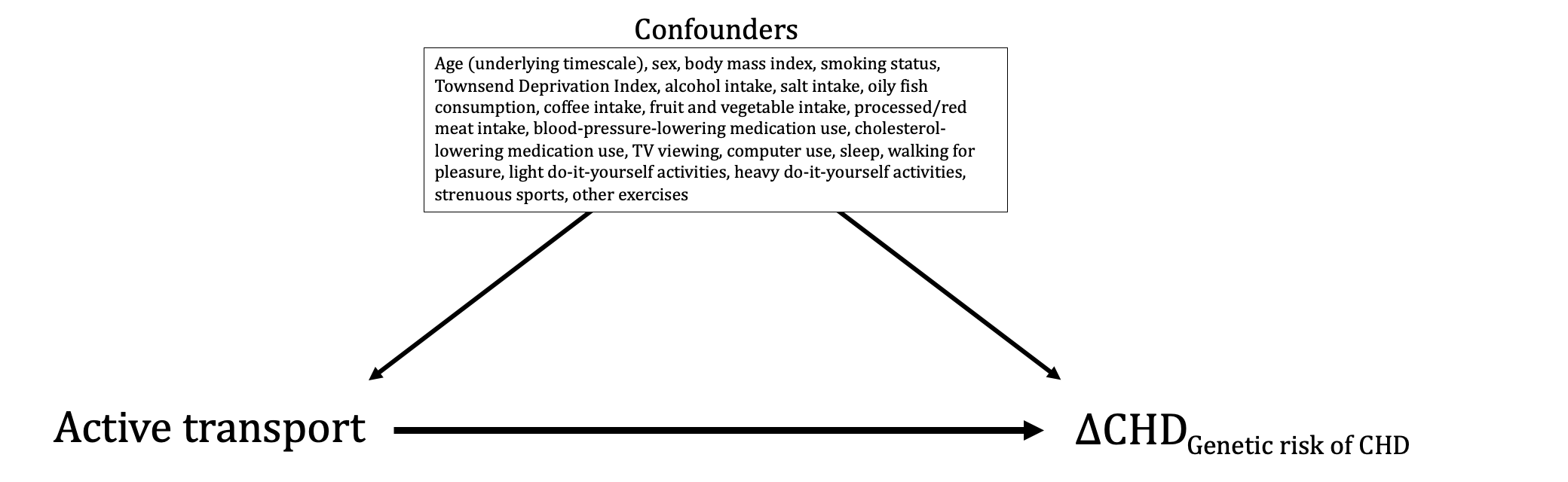


# Supplemental Figure 3. An interaction directed acyclic graph (IDAG) describing the conceptual framework for the interplay of active transport and genetic risk of coronary heart disease (CHD) in relation to risk of CHD.^1^

Reference

1. Nilsson, A., Bonander, C., Strömberg, U., and Björk, J. “A directed acyclic graph for interactions,” Int J Epidemiol, vol. 50, no. 2, pp. 613-619, 2021, doi: 10.1093/ije/dyaa211


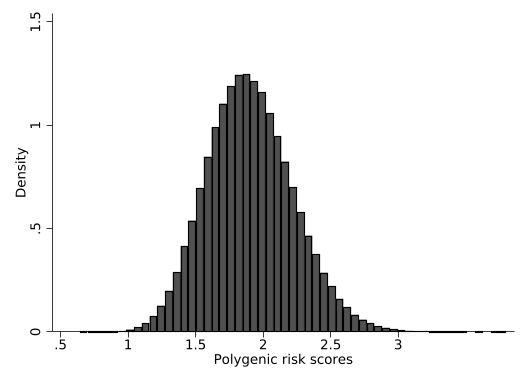


# Supplemental Figure 4. Distribution of the calculated polygenic risk score (PRS) for coronary heart disease using 46 lead SNPs (from 46 loci) which were genome-wide significant (p-value: 5×10^-8^) and in low linkage disequilibrium (r^2^<0.001).
